# Cell‐Type‐Dependent Metabolic Compensation Preserves Photoreceptor Survival Through Pyruvate Kinase Isoform Balance

**DOI:** 10.1096/fj.202505064R

**Published:** 2026-03-31

**Authors:** Ammaji Rajala, Larissa J. Trevino, Tyler M. Black, Raju V. S. Rajala

**Affiliations:** ^1^ Department of Ophthalmology University of Oklahoma Health Sciences Center Oklahoma City Oklahoma USA; ^2^ OU Health Dean McGee Eye Institute Oklahoma City Oklahoma USA; ^3^ Departments of Biochemistry and Physiology University of Oklahoma Health Sciences Center Oklahoma City Oklahoma USA; ^4^ Department of Cell Biology University of Oklahoma Health Sciences Center Oklahoma City Oklahoma USA

**Keywords:** LDHA, neurodegeneration, photoreceptors, PKM1, PKM2, retinal metabolism

## Abstract

Photoreceptors depend on aerobic glycolysis to meet the high biosynthetic demand required for continuous outer segment renewal. Disruption of this metabolic program is increasingly recognized as a contributor to retinal degeneration; however, the coordinated roles of key glycolytic enzymes across retinal cell types remain incompletely understood. Pyruvate kinase M2 (PKM2) and lactate dehydrogenase A (LDHA) are central regulators of aerobic glycolysis; however, the mechanisms by which their interplay supports retinal homeostasis remain unclear. Here, we investigated the effects of selectively deleting LDHA alone or in combination with PKM2 in retinal neurons. Rod‐specific deletion of LDHA, as well as combined deletion of LDHA and PKM2 in rods, led to progressive photoreceptor degeneration, accompanied by structural disorganization and functional impairment. Loss of LDHA reduced PKM2 expression and induced compensatory upregulation of PKM1; however, PKM1 levels did not reach those of PKM2, correlating with increased susceptibility to degeneration. In contrast, deletion of both LDHA and PKM2 throughout the retina led to robust PKM1 induction to levels comparable to those of PKM2 and was associated with preservation of retinal structure and function. Translating ribosome affinity purification demonstrated that LDHA, LDHB, PKM1, and PKM2 are expressed across multiple retinal cell types, and metabolic analyses revealed that non‐rod neurons contribute substantially to retinal lactate production. We propose a PKM isoform balance threshold model in which retinal outcome depends on the level of PKM1 compensation following PKM2 loss. When PKM1 reaches levels comparable to PKM2, retinal structure and function are preserved; insufficient compensation results in degeneration. These findings highlight cell–type–dependent metabolic compensation and pyruvate kinase isoform balance as key determinants of retinal integrity and photoreceptor survival.

## Graphical abstract

We investigated the roles of PKM2, PKM1, and LDHA in retinal metabolism and structural integrity. In the normal retina, these enzymes are maintained at defined levels that support photoreceptor function. Rod‐specific deletion of LDHA, alone or with PKM2, caused age‐dependent degeneration, as PKM1 induction did not reach levels comparable to PKM2. In contrast, pan‐retinal deletion elevated PKM1 to PKM2‐equivalent levels, restored glycolytic flux, and preserved retinal structure. These findings support a PKM isoform balance threshold model governing retinal resilience.

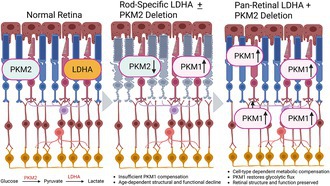

## 1 Introduction

Photoreceptor cells operate under a continuous anabolic burden due to the daily renewal of their outer segments, a process that requires substantial lipid and protein synthesis [1, 2, 3, 4, 5]. To support these demands, photoreceptors depend on aerobic glycolysis, a metabolic program commonly associated with rapidly dividing tumor cells [6, 7]. In this pathway, pyruvate kinase M2 (PKM2) and lactate dehydrogenase A (LDHA) play key roles. PKM2, in its low‐activity dimeric form, allows glycolytic intermediates to flow into biosynthetic pathways, while LDHA converts pyruvate to lactate and regenerates NAD^+^, enabling glycolysis to continue at high flux [8, 9]. Although this arrangement is well understood in cancer metabolism, its importance in postmitotic photoreceptors is only beginning to emerge.

When mitochondrial shuttle systems responsible for oxidizing cytosolic NADH reach capacity, LDHA becomes critical for maintaining NAD^+^ levels [9]. Photoreceptors, with their intense metabolic activity and daily biosynthetic requirements, are likely operating near this threshold, which explains their strong LDHA expression. PKM2 further contributes by linking glycolytic output to the synthesis of new cellular components. However, how these enzymes function together to maintain photoreceptor health and how their loss influences retinal physiology remains unclear.

In this study, we examined the roles of LDHA and PKM2 in photoreceptor metabolism and survival using genetic models targeting rods and the entire retina. We found that loss of LDHA, and the combined loss of LDHA and PKM2 in rods, leads to progressive, age‐dependent photoreceptor degeneration, whereas deleting both enzymes in all retinal neurons unexpectedly preserves retinal structure and function. A central finding is that LDHA loss reduces PKM2 expression and induces PKM1 upregulation; however, this compensatory response differs between rods and other retinal cell types. We propose a PKM isoform balance threshold model in which retinal survival depends on the degree of PKM1 compensation following PKM2 loss. When PKM1 rises to levels comparable to PKM2, retinal structure and function are preserved, whereas inadequate compensation results in degeneration. These results reveal cell‐type‐specific metabolic adaptation in the retina and identify PKM isoform balance as an essential factor in maintaining photoreceptor integrity.

## 2 Materials and Methods

### 2.1 Animals

All animal procedures complied with the ARVO Statement for the Use of Animals in Ophthalmic and Vision Research and the National Institutes of Health Guide for the Care and Use of Laboratory Animals. All experimental protocols were approved by the Institutional Animal Care and Use Committee (IACUC) at the University of Oklahoma Health Sciences Center. Breeding stocks for floxed Pkm2 (Jax #024048), Chx10‐Cre (Jax #005105), floxed Ldha (Stock No: 030112), and RiboTag (Jax #011029) mice were purchased from The Jackson Laboratory (Bar Harbor, ME). Rhodopsin‐Cre (i75Cre) mice were kindly provided by Dr. Ching‐Kang Jason Chen at Baylor College of Medicine (Houston, TX). Mice were bred and maintained in our institutional animal facility under a controlled 12h light/dark cycle (40–60 lx). All animals were screened for the *rd1* and *rd8* retinal degeneration mutations and were confirmed to be negative. To minimize experimental bias, mice were randomly assigned to groups balanced for sex, age, and genetic background. Litters were combined to avoid litter‐specific variability.

### 2.2 Affinity Purification of Translated mRNAs From Rod, Cone, Müller, RGC, and RPE Cells

RiboTag mice carrying an HA‐tagged ribosomal protein (Rpl22^HA^) were crossed with rod‐specific rhodopsin‐Cre, cone‐specific cone‐opsin‐Cre, Müller cell–specific Rax‐Cre^ERT2^, and tetracycline‐inducible RPE‐specific VMD2‐Cre lines. To isolate retinal ganglion cell (RGC) transcripts, AAV2‐CMV‐Cre (Vector Biolabs) was injected intravitreally into floxed Rpl22 mice, and retinas were collected 2‐weeks later for mRNA extraction. This enabled Cre‐dependent incorporation of the HA epitope into ribosomes, thereby allowing the isolation of actively translating mRNAs from rod photoreceptors, cone photoreceptors, Müller cells, RGC, and RPE cells. Rax‐Cre^ERT2^ activity was induced by administering 1 mg tamoxifen by gavage every other day for three doses. VMD2‐Cre was induced by doxycycline gavage at 0.4 mg/g body weight for two consecutive days. Polyribosomes bound to mRNA were then immunoprecipitated from each targeted cell type, and the expression of *Ldha, Ldhb*, and *Rpl38* was assessed by qRT‐PCR (Table S1). *Ldha* and *Ldhb* transcript levels were normalized to *Rpl38*.

### 2.3 Pyruvate Kinase Activity

Pyruvate kinase (PK) activity was measured using a lactate dehydrogenase (LDH)–coupled enzyme assay [10]. The reaction contained mouse retinal lysate and enzyme buffer consisting of 50 mM Tris–HCl (pH 7.4), 100 mM KCl, 5 mM MgCl_2_, 1 mM ADP, 0.5 mM phosphoenolpyruvate (PEP), 0.2 mM NADH, and 8 U of LDH. PK activity was monitored spectrophotometrically by measuring the decrease in absorbance at 340 nm, reflecting NADH oxidation.

### 2.4 Lactate Efflux

Lactate efflux was measured as previously described [11]. Retinas were incubated ex vivo in Krebs–Ringer–Bicarbonate (KRB) buffer containing 5 mM glucose or other specified additives. After 30 min, lactate released into the medium was quantified using the Lactate Reagent (Trinity Biotech, Bray Co. Wicklow, Ireland) according to the manufacturer's instructions.

### 2.5 Mitochondrial Isolation

Mitochondria were isolated from the retina using a previously described method for cells [12], with the following modifications. Briefly, retinas were harvested and placed in cold mitochondrial isolation medium (MIM: 300 mM sucrose, 10 mM HEPES, 0.2 mM EDTA, and 1 mg/mL BSA, pH 7.4). The retinas were homogenized using a plastic handheld homogenizer with 20 strokes. The homogenate was centrifuged at 700 × g for 7 min at 4°C to remove nuclei and debris. The supernatant was transferred to a new tube, and the pellet was re‐homogenized in fresh MIM to recover additional mitochondria. Both supernatants were pooled and centrifuged at 10000 × g for 10 min at 4°C to obtain the mitochondrial pellet. The mitochondrial pellets were washed with cold MIM lacking BSA, and total protein concentration was measured using the Bradford protein assay.

### 2.6 Statistical Analysis

Sample sizes were selected based on power calculations. All analyses were performed using GraphPad Prism version 7.3, and no data points were removed. Statistical tests were selected based on the experimental design. Before analysis, each dataset was assessed for normality using the Anderson–Darling, D'Agostino–Pearson, Shapiro–Wilk, and Kolmogorov–Smirnov tests. For datasets that did not meet the normality requirements, nonparametric tests were used, specifically multiple Mann–Whitney *U* tests. To control for multiple comparisons and reduce the false discovery rate, the two‐stage Benjamini–Krieger–Yekutieli method was applied with a *Q* value of 1%. For normally distributed data, comparisons between two groups were performed using parametric tests with Welch's correction to account for unequal variances. Significance was assessed based on *p*‐values. One‐way ANOVA was used to compare three or more independent groups, and two‐way ANOVA was used when evaluating the effects of two independent variables.

### 2.7 Other Methods

Electroretinography (ERG), tissue histology, immunoblotting, and immunohistochemical staining were performed following previously established protocols [13, 14]. The primers and antibodies used in this study are listed in Tables S1 and S2, respectively.

## 3 Results

### 3.1 Expression of LDHA Isoforms in Various Retinal Cell Types

**FIGURE 1:**
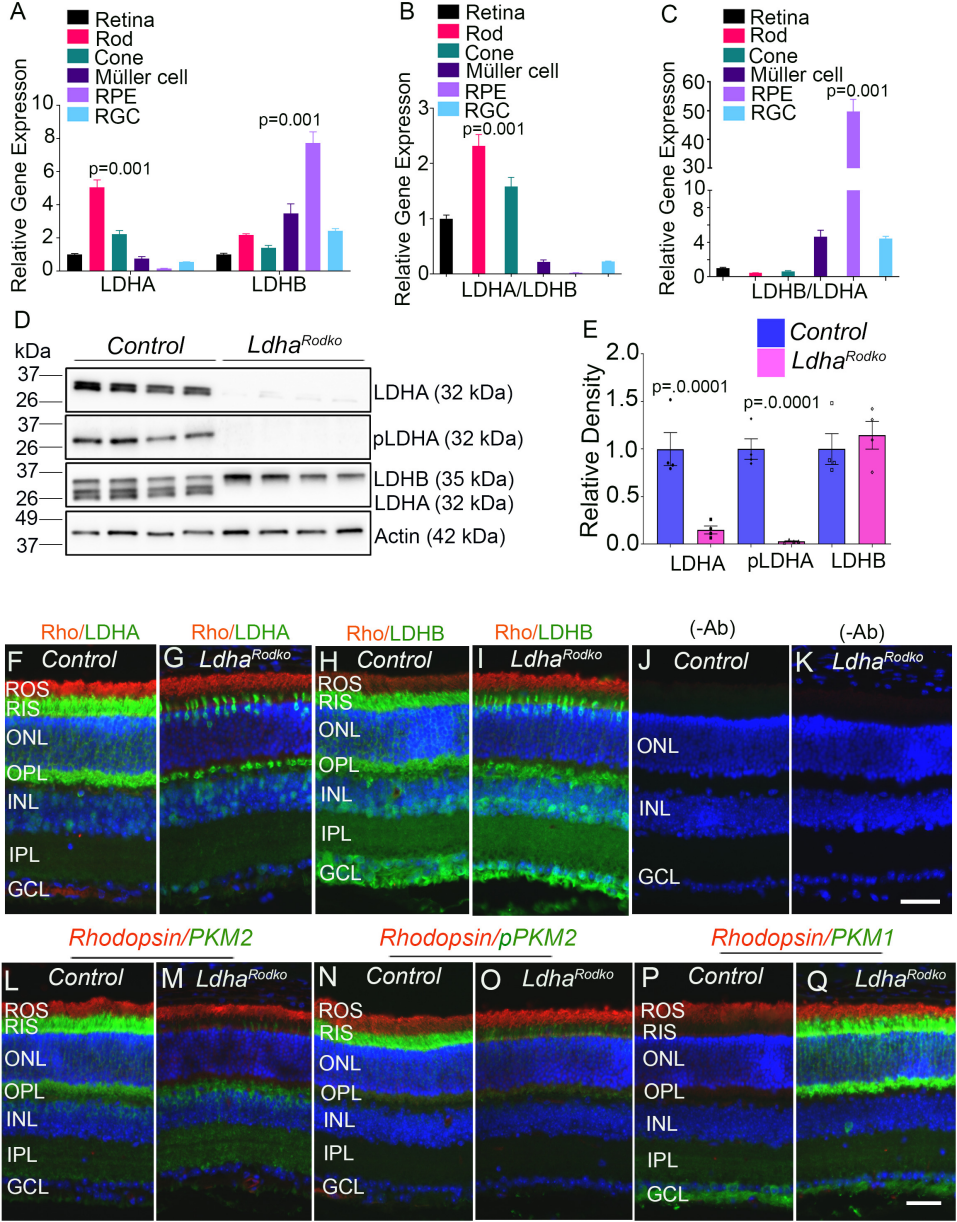
Expression and localization of LDHA and LDHB in the retina and functional consequences of rod‐specific *Ldha* deletion. Equal amounts of mRNA from the retina, rod photoreceptors, cone photoreceptors, Müller cells, RPE, and RGCs were analyzed by qRT‐PCR to measure *Ldha* and *Ldhb* expression, with values normalized to *Rpl38* (A). (B) shows the LDHA/LDHB ratio, and (C) shows the LDHB/LDHA ratio. The mRNA levels are presented as mean ± SEM (*n* = *3*). Four week old control and *Ldha* ^*Rodko*^ mouse retina lysates were immunoblotted using antibodies against LDHA and LDHB (D), and their levels were normalized to Actin (E). Data are mean ± SEM (*n* = *3*). Retina sections from 4 week old control and *Ldha* ^*Rodko*^ mice were immunostained with Rhodopsin/LDHA (F, G), Rhodopsin/LDHB (H, I), Rhodopsin/PKM2 (L, M), Rhodopsin/pPKM2 (N, O), and Rhodopsin/PKM1 (P, Q) antibodies. (J) and (K) show omission of primary antibodies. ROS, rod outer segments; RIS, rod inner segments; ONL, outer nuclear layer; OPL, outer plexiform layer; INL, inner nuclear layer; IPL, inner plexiform layer; GCL, ganglion cell layer. Scale bar = 50 μm.

Using Translation Ribosome Affinity Purification (TRAP), we have isolated mRNA from total retina, rod, cone, Müller cells, retinal pigment epithelium (RPE), and retinal ganglion cells (RGC) after mating the RiboTag‐Rpl22 mice with respective cell‐type‐Cre mice [13]. Quantitative real‐time PCR with the LDHA primers shows that rod and cone photoreceptor cells mainly express LDHA isoforms compared to the total retina, Müller cells, and RGC (Figure 1A). The expression of LDHB is significantly enriched in RPE compared to Müller cells, rod, cone, and RGC (Figure 1A). These findings were further supported by calculating the LDHA/LDHB and LDHB/LDHA ratios. The results confirmed that LDHA is predominantly expressed in rods and cones (Figure 1B), whereas LDHB is primarily expressed in the RPE, followed by Müller glia and retinal ganglion cells (Figure 1C).

### 3.2 Generation and Characterization of Rod‐Specific LDHA KO Mice

To determine the functional role of LDHA in rod photoreceptor cells, we generated rod‐specific LDHA knockout (*Ldha* ^*Rodko*^) mice by breeding rhodopsin‐Cre mice with LDHA floxed (control, *Ldha* ^*flox/flox*^) mice. One month old *Ldha* ^*Rodko*^ and control mouse retina lysates were immunoblotted with antibodies against LDHA, pLDHA (Tyr10), LDHB, and actin. The results indicate that more than 90% of LDHA was deleted in the *Ldha* ^*Rodko*^ mice compared to control mice (Figure 1D,E). Furthermore, consistent with the loss of LDHA protein in *Ldha* ^*Rodko*^ mice, phosphorylation of LDHA was also absent in these retinas (Figure 1D,E). The LDHB antibody recognizes both LDHA and LDHB. We observed a loss of LDHA when probed with the LDHB antibody (Figure 1D,E). LDHB protein levels appear higher in *Ldha* ^*Rodko*^ mice than in control mice; however, the difference is not statistically significant (Figure 1D,E).

Immunohistochemical analysis further reveals a complete loss of LDHA expression in the photoreceptors of *Ldha* ^*Rodko*^ mice compared to control mice (Figure 1F,G). In contrast, rhodopsin expression appears to be normal in both genotypes (Figure 1F,G). The residual LDHA expression observed in the outer segment region of *Ldha* ^*Rodko*^ mice is from cone photoreceptors and their cell bodies (Figure 1G). LDHB expression is detected throughout the retina in control mice (Figure 1H), as the antibody used recognizes both LDHA and LDHB. In *Ldha* ^*Rodko*^ mice, staining is confined to the inner retinal layers (Figure 1I). Because this antibody does not distinguish between LDHA and LDHB, cone staining in *Ldha* ^*Rodko*^ mice cannot be definitively attributed to either isoform. Together, these findings indicate that LDHA is predominantly expressed in photoreceptors, whereas LDHB is expressed primarily in the inner retina.

### 3.3 Effect of Rod‐Specific LDHA Loss on Metabolic Enzyme Expression

**FIGURE 2:**
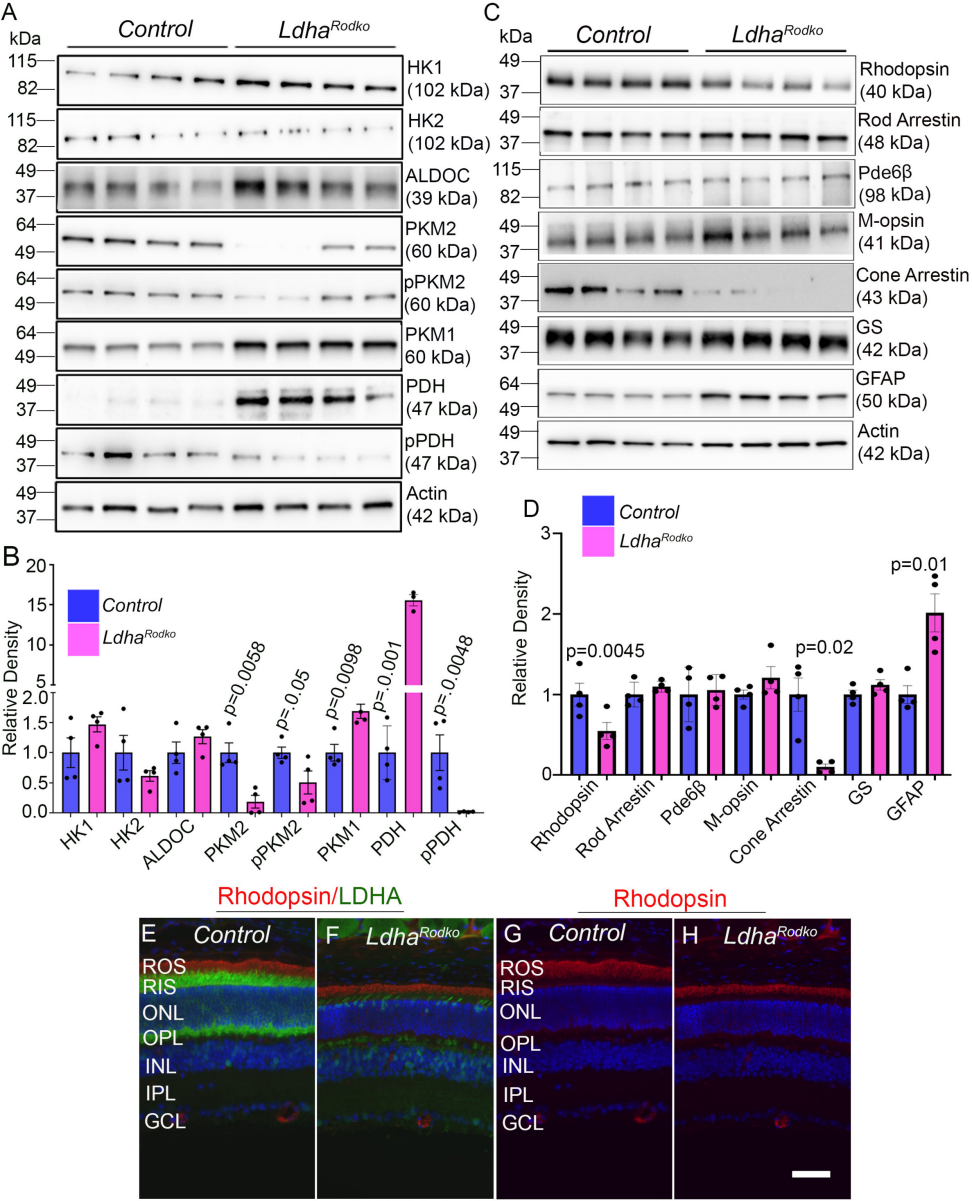
Characterization of rod‐specific LDHA knockout mice. Retina lysates from 4 week old control and *Ldha* ^*Rodko*^ mice were immunoblotted with antibodies against HK1, HK2, ALDOC, PKM2, pPKM2, PKM1, PDH, pPDH, and Actin (A), and their levels were normalized to Actin (B). Data are mean ± SEM (*n* = *4*). Retinal lysates from 20 week old control and *Ldha* ^*Rodko*^ mice were immunoblotted with antibodies against rhodopsin, rod arrestin, Pde6β, M‐opsin, cone arrestin, GS, GFAP, and Actin (C), and the levels were normalized to Actin (D). Data are mean ± SEM (*n* = *4*). Retina sections from 24 week old control and *Ldha* ^*Rodko*^ mice were immunostained with Rhodopsin/LDHA (E, F) and Rhodopsin (G, H) antibodies. Scale bar = 50 μm. Note: We used the same Actin blot in Figures 1D and 2A because both panels were generated from the same samples of the same age, for ease of comparison.

Immunohistochemistry shows loss of PKM2 expression (Figure 1L,M) and its phosphorylation (Figure 1N,O), along with a compensatory increase in PKM1 (Figure 1P,Q; Figure S1 shown at lower saturation) in rod photoreceptors of the *Ldha* ^*Rodko*^ mouse retina compared with controls. The rhodopsin expression appears to be normal in *Ldha* ^*Rodko*^ mice compared to control mice (Figure 1L–Q). We analyzed the protein levels of hexokinase 1 (HK1), hexokinase 2 (HK2), aldolase C (ALDOC), PKM2, phosphorylated PKM2 (pPKM2‐Y105), PKM1, pyruvate dehydrogenase (PDH), and phosphorylated PDH (pPDH), and normalized their levels to actin. The results show increased levels of HK1 in *Ldha* ^*Rodko*^ mice compared with control mice; however, the difference was not statistically significant (Figure 2A,B). Interestingly, the loss of LDHA in rods results in significantly decreased levels of PKM2 and its phosphorylation (pPKM2), as well as increased levels of PKM1 (Figure 2A,B). We also found increased levels of ALDOC, which regulates Fructose‐1,6‐bisphosphate (Fru‐1,6‐BP), an allosteric activator of PKM2 [3], in *the Ldha* ^*Rodko*^ mouse retina compared with the control mouse retina (Figure 2A,B). We also found significantly higher levels of PDH and lower levels of its phosphorylation (pPDH) in *Ldha* ^*Rodko*^ mice compared with the control mouse retina (Figure 2A,B). Phosphorylation of PDH by pyruvate dehydrogenase kinase (PDK) [15], which decreases PDH affinity toward oxidative decarboxylation of pyruvate with the formation of acetyl‐CoA [16]. These observations suggest that reduced PDH phosphorylation, together with increased PDH levels following LDHA loss in rods, may promote a shift toward enhanced oxidative phosphorylation. Twenty week old *Ldha* ^*Rodko*^ and control mouse retinas were immunoblotted with rhodopsin, rod arrestin, Pde6β, M‐opsin, cone arrestin, glutamine synthetase (GS), and glial fibrillary acidic protein (GFAP), and their expression was normalized to actin. The results indicate significantly lower levels of rhodopsin and cone arrestin in the *Ldha* ^*Rodko*^ mouse retina than in the control retina (Figure 2C,D), suggesting photoreceptor degeneration. GFAP levels were significantly higher in *Ldha* ^*Rodko*^ mouse retinas than in control mouse retinas (Figure 2C,D), indicating reactive gliosis, a response typically observed following injury or disease. Consistent with the immunoblotting, rhodopsin expression and ONL thickness are decreased in the 20 week old *Ldha* ^*Rodko*^ mouse retina compared to the control retina (Figure 2E–H).

### 3.4 Age‐Dependent Changes in Retinal Structure and Function Following Rod‐Specific Deletion of LDHA

**FIGURE 3:**
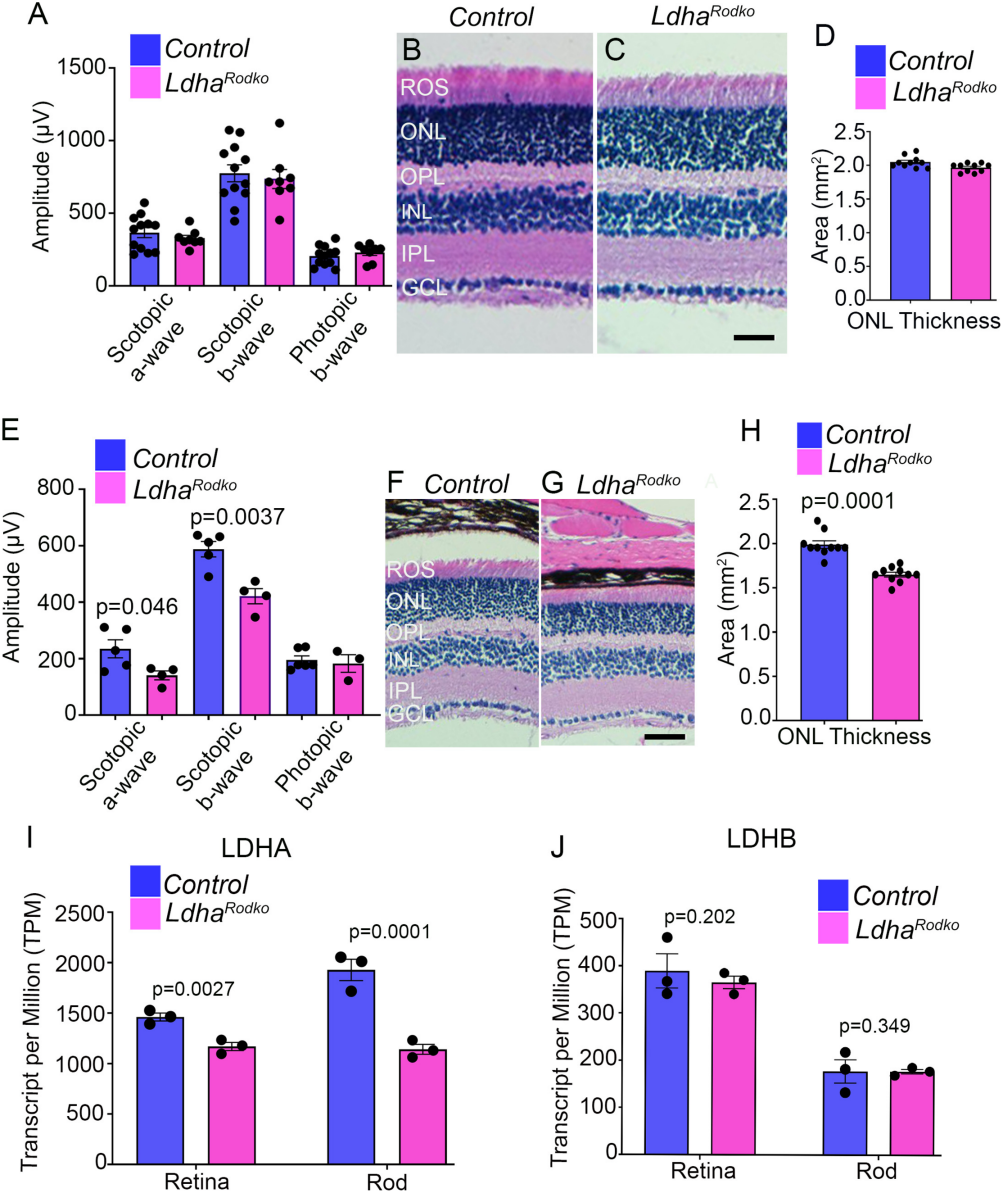
Rod‐specific deletion of LDHA leads to progressive functional decline and structural retinal changes. Scotopic a‐ and b‐wave amplitudes and photopic b‐wave amplitudes were measured in 4 week old control and *Ldha* ^*Rodko*^ mice (A). Data are shown as mean ± SEM (*n* = *12*). Four week old control and *Ldha* ^*Rodko*^ mouse retina sections were stained with hematoxylin and eosin and examined for morphology (B, C). Scale bar = 50 μm. Quantification of ONL thickness was performed using NIH ImageJ software (D). Data are mean ± SEM (*n* = *10*). Scotopic a‐wave, scotopic b‐wave, and photopic b‐wave analyses were performed on 24 week old control and *Ldha* ^*Rodko*^ mice (E). Data are mean ± SEM (*n* = *5*). Twenty‐four week old control and *Ldha* ^*Rodko*^ mouse retina sections were stained with hematoxylin and eosin and examined for morphology (F, G). Scale bar = 50 μm. Quantification of ONL thickness was performed using NIH ImageJ software (H). Data are mean ± SEM (*n* = *10*). Transcript per million mapped reads (TPM) of LDHA (I) and LDHB (J) are measured from the rod and retina of control and *Ldha* ^*Rodko*^ mice. Data are mean ± SEM (*n* = *3*).

The retinal function of 4 week old *Ldha* ^*Rodko*^ mice showed no significant differences in scotopic a‐wave, scotopic b‐wave (rod responses), or photopic b‐wave (cone responses) amplitudes compared to control mice (Figure 3A). Consistent with the unchanged ERG results, retinal morphology in *Ldha* ^*Rodko*^ mice was comparable to that of control mice, with no significant structural differences observed (Figure 3B,C). Quantification of ONL thickness further confirmed the absence of detectable degeneration (Figure 3D). In 20 week old *Ldha* ^*Rodko*^ mice, scotopic a‐wave and scotopic b‐wave amplitudes (rod responses) were significantly reduced (Figure 3E). In contrast, photopic b‐wave amplitudes (cone responses) remain unchanged compared to control mice (Figure 3E). Histological analysis showed thinning of the outer segments and a reduction in outer nuclear layer thickness in *Ldha* ^*Rodko*^ mice relative to controls (Figure 3F–H). Together, these findings indicate age‐dependent retinal degeneration in mice lacking LDHA in rod photoreceptors.

### 3.5 Selective Reduction of *Ldha* Transcripts but Not *Ldhb* in *Rod Photoreceptor Cells*

RNA‐seq analysis revealed a significant decrease in *Ldha* expression in *Ldha* ^*Rodko*^ rods (Figure 3I). The baseline expression of *Ldha* in control rods is approximately 1927 transcripts per million (TPM), which decreases by about 41% to ~1140 TPM in *Ldha* ^*Rodko*^ rods (Figure 3I). The baseline expression of *Ldhb* in rods is approximately 180 TPM and remains unchanged in *Ldha* ^*Rodko*^ mice (Figure 3J). In the retina, *Ldhb* expression is approximately 380 TPM and remains unchanged in *Ldha* ^*Rodko*^ mice (Figure 3I). Because *Ldha* is predominantly expressed in rod photoreceptors, its TPM levels appear much lower when measured at the whole‐retina level (Figure 3J).

### 3.6 Generation and Characterization of Rod‐Specific PKM2/LDHA Double Knockout Mice

**FIGURE 4:**
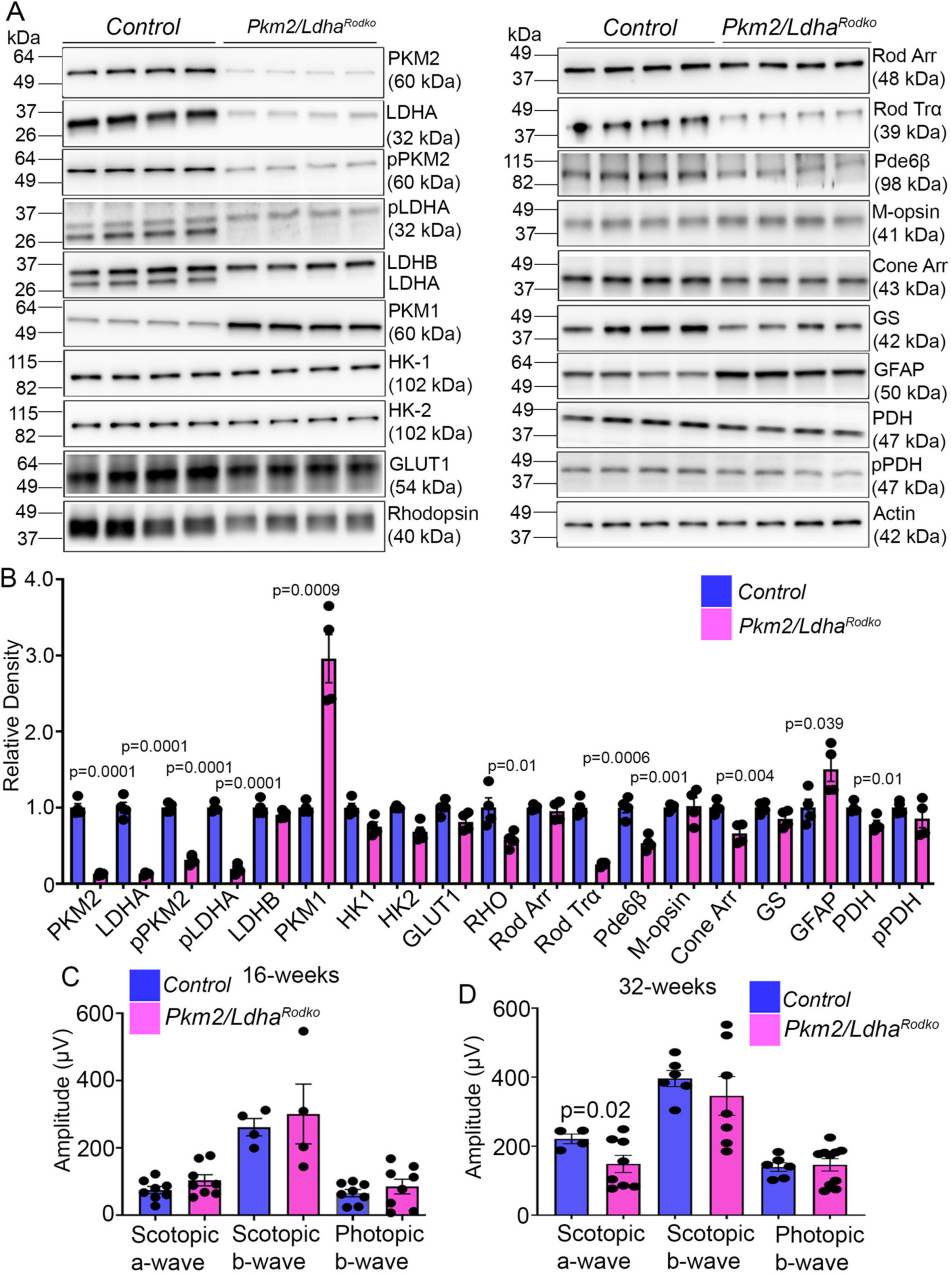
Characterization of the biochemical and functional effects of PKM2 and LDHA loss in rod photoreceptors. Retina lysates from 4 week old control and *Pkm2*/*Ldha* ^*Rodko*^ mice were immunoblotted with antibodies against PKM2, LDHA, pPKM2, pLDHA, LDHB, PKM1, HK1, HK2, GLUT1, and Actin (A), and their levels were normalized to Actin (B). Data are mean ± SEM, (*n* = *4*). Retinal lysates from 20 week old control and *Pkm2/Ldha* ^*Rodko*^ mice were immunoblotted with antibodies against rhodopsin, rod arrestin, Pde6β, M‐opsin, cone arrestin, GS, GFAP, PDH, pPDH, and Actin (A), and the levels were normalized to Actin (B). Data are mean ± SEM, (*n* = *4*). Scotopic a‐ and b‐wave amplitudes and photopic b‐wave amplitudes were measured in 16 week old (C) and 32 week old (D) control and *Pkm2Ldha* ^*Rodko*^ mice. Data are mean ± SEM (*n* = *8*).

We generated rod‐specific PKM2/LDHA double knockout (*Pkm2/Ldha* ^*Rodko*^) mice by crossing *Pkm2*‐ and *Ldha*‐floxed mice with mice expressing Cre recombinase under the control of the rhodopsin promoter. Immunoblotting analysis of 1 month old control (*Pkm2/Ldha* ^*flox/flox*^) and *Pkm2/Ldha* ^*Rodko*^ mouse retina shows significantly decreased levels of PKM2 and its phosphorylation (pPKM2), LDHA and its phosphorylation (pLDHA), and increased levels of PKM1 in *Pkm2/Ldha* ^*Rodko*^ mouse retina compared to control mouse retina (Figure 4A,B). There was no change in the levels of hexokinase 1 (HK1), HK2, and glucose transporter 1 (GLUT1) between control and *Pkm2/Ldha* ^*Rodko*^ mouse retina (Figure 4A,B). Twenty‐four week old control and *Pkm2/Ldha* ^*Rodko*^ mouse retinas were immunoblotted with antibodies against rhodopsin, rod arrestin, rod transducin alpha (Trα), Pde6β, M‐opsin, cone arrestin, GS, GFAP, PDH, and pPDH, and their levels were normalized to actin. *Pkm2/Ldha* ^*Rodko*^ retinas showed significantly reduced levels of rhodopsin, Trα, Pde6β, cone arrestin, and PDH, along with increased GFAP compared to control retinas (Figure 4A,B).

Consistent with the immunoblot analysis, immunohistochemistry further confirms the loss of PKM2 (Figure S2A and D) and LDHA (Figure S2B and E), and the increased PKM1 expression (Figure S2C and F) in the *Pkm2/Ldha* ^*Rodko*^ mouse retina compared with the control retina. In the absence of LDHA, there was no upregulation of LDHB in the *Pkm2/Ldha* ^*Rodko*^ mouse retina (Figure S3A,B). The phosphorylation of PKM2 was absent in *Pkm2/Ldha* ^*Rodko*^ mouse retina compared to the control retina (Figure S3C,D).

### 3.7 Age‐Dependent Alterations in Retinal Structure and Function in Rod‐Specific Deletion of PKM2 and LDHA

**FIGURE 5:**
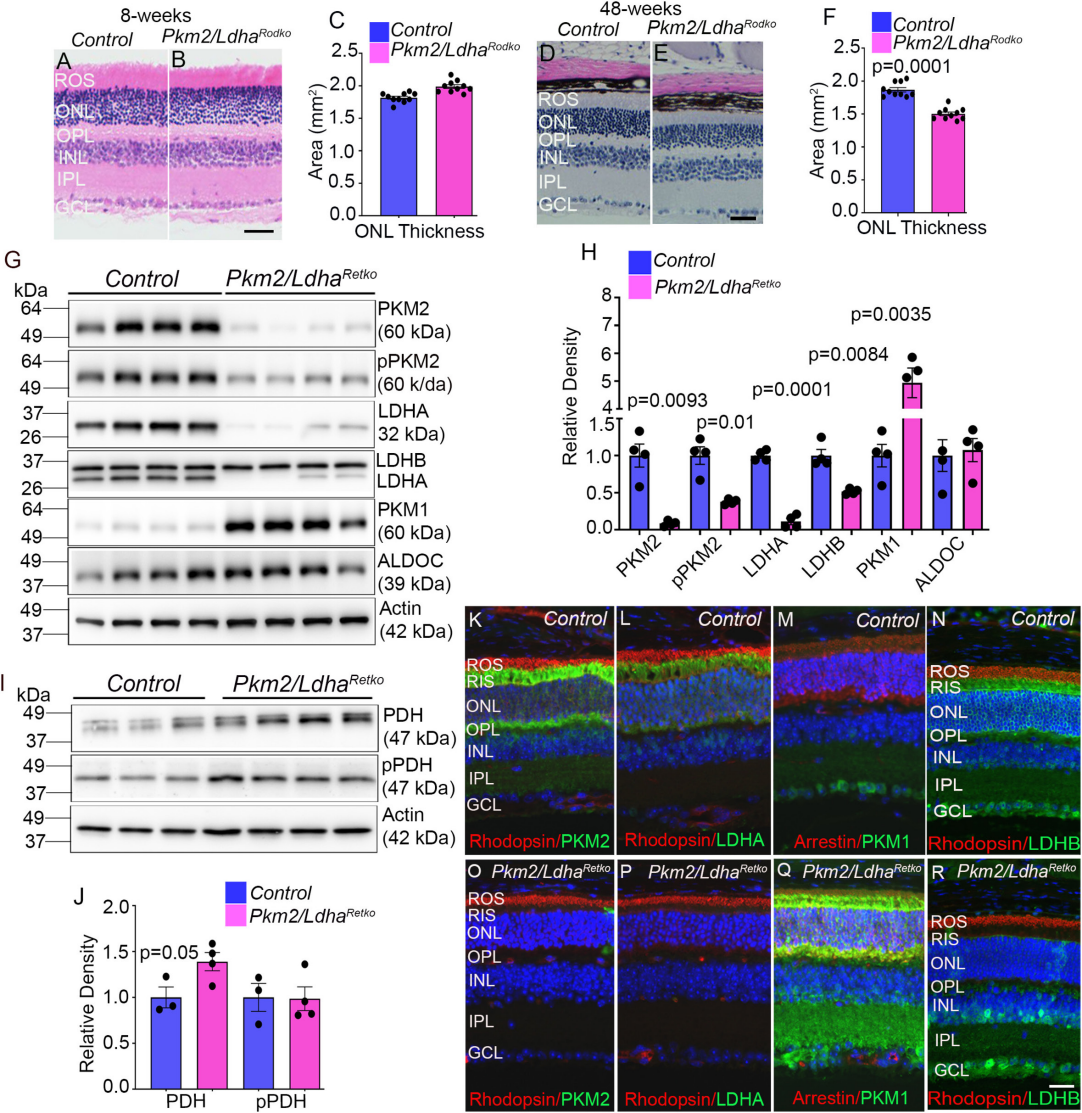
Structural and biochemical characterization of rod‐specific and pan‐retinal PKM2 and LDHA deletion. Retinal sections from 8 week old (A, B) and 48 week old (D, E) control and *Pkm2*/*Ldha* ^*Rodko*^ mice were stained with hematoxylin and eosin and examined for morphology. Scale bar = 50 μm. ONL thickness at 8 weeks (C) and 48 weeks (F) was quantified using NIH ImageJ software. Data are mean ± SEM (*n* = *10*). Retina lysates from 4 week old control and *Pkm2*/*Ldha* ^*Retko*^ mice were immunoblotted with antibodies against PKM2, pPKM2, LDHA, LDHB, PKM1, ALDOC, and Actin (G), and their levels were normalized to Actin (H). Data are mean ± SEM (*n* = *4*). Retina sections from control (K‐N) and *Pkm2Ldha* ^*Retko*^ mice (O‐R) were immunostained with Rhodopsin/PKM2 (K, O), Rhodopsin/LDHA (L‐P), Arrestin/PKM1 (M, Q), and Rhodopsin/LDHB (N, R) antibodies. Please note that the LDHB antibody recognizes both LDHA and LDHB isoforms (N). In pan‐retina PKM2/LDHA KO tissues, LDHB staining is detected only in the inner retina and not in the outer retina (R), indicating that LDHB is predominantly expressed in the inner retina, whereas LDHA is primarily expressed in the outer retina.

Sixteen week old *Pkm2/Ldha* ^*Rodko*^ mice showed no significant differences in scotopic a‐wave, scotopic b‐wave (rod function), or photopic b‐wave (cone function) amplitudes compared with control mice (Figure 4C). At 32 weeks of age, *Pkm2/Ldha* ^*Rodko*^ mice showed a significant decrease in scotopic a‐wave amplitude, with no changes in scotopic b‐wave or photopic b‐wave amplitudes compared to control mice (Figure 4D). At 8 weeks, histological examination of H&E‐stained retinal sections showed no change in ONL thickness (Figure 5A–C). However, by 48 weeks, significant thinning of the outer nuclear layer was observed in *Pkm2/Ldha* ^*Rodko*^ mice compared with controls (Figure 5D–F), indicating progressive retinal degeneration.

### 3.8 Generation and Characterization of Retina‐Specific PKM2/LDHA Double Knockout Mice

We generated retina‐specific PKM2/LDHA double knockout (*Pkm2/Ldha* ^*Retko*^) mice by crossing *Pkm2*‐ and *Ldha*‐floxed mice with mice expressing Cre recombinase under the control of Chx10 promoter, a homeobox gene expressed in all retinal progenitor cells [14]. Retinal lysates from 1 month old Pkm2/*Ldha* ^*Retko*^ and control mice were immunoblotted with antibodies against PKM2, pPKM2, LDHA, LDHB, and ALDOC, and band intensities were normalized to actin. More than 90% of PKM2 and LDHA were eliminated in *Pkm2*/*Ldha* ^*Retko*^ retinas compared with controls (Figure 5G,H). As expected, the loss of PKM2 protein was accompanied by a significant reduction in PKM2 phosphorylation in these mice (Figure 5G,H). PKM1 levels were significantly higher in *Pkm2/Ldha* ^*Retko*^ retinas than in controls (Figure 5G,H). Because the LDHB antibody detects both LDHA and LDHB, loss of LDHA was also evident with this antibody (Figure 5G,H). LDHB protein levels were also significantly reduced in *Pkm2/Ldha* ^*Retko*^ retinas than in controls (Figure 5G,H). Retinal lysates were immunoblotted with antibodies against PDH and phospho‐PDH. The results show a significant increase in total PDH protein levels in Pkm2/*Ldha* ^*Retko*^ mice compared with controls, whereas PDH phosphorylation levels remained unchanged between the two groups (Figure 5I,J).

Immunohistochemical analysis further reveals a complete loss of PKM2 (Figure 5K,O) and LDHA (Figure 5L,P) expression in the photoreceptors of *Pkm2/Ldha* ^*Retko*^ mice compared to control mice. In contrast, rhodopsin expression appears to be normal in both genotypes (Figure 5K,L,O,P). Consistent with the immunoblot results, we observed a compensatory increase in PKM1 expression in rod photoreceptors, along with strong PKM1 staining in the inner plexiform layer of the *Pkm2/Ldha* ^*Retko*^ mouse retina compared with controls (Figure 5M,Q). The LDHB antibody recognizes both LDHA and LDHB isoforms. In control retinas, staining is observed throughout the retina, from the rod inner segments to the ganglion cell layer (Figure 5N). In *Pkm2/Ldha* ^*Retko*^ mice, staining is restricted to the inner retina and absent from the outer retina (Figure 5R), indicating that LDHA is predominantly expressed in the outer retina, whereas LDHB is localized to the inner retina.

**FIGURE 6:**
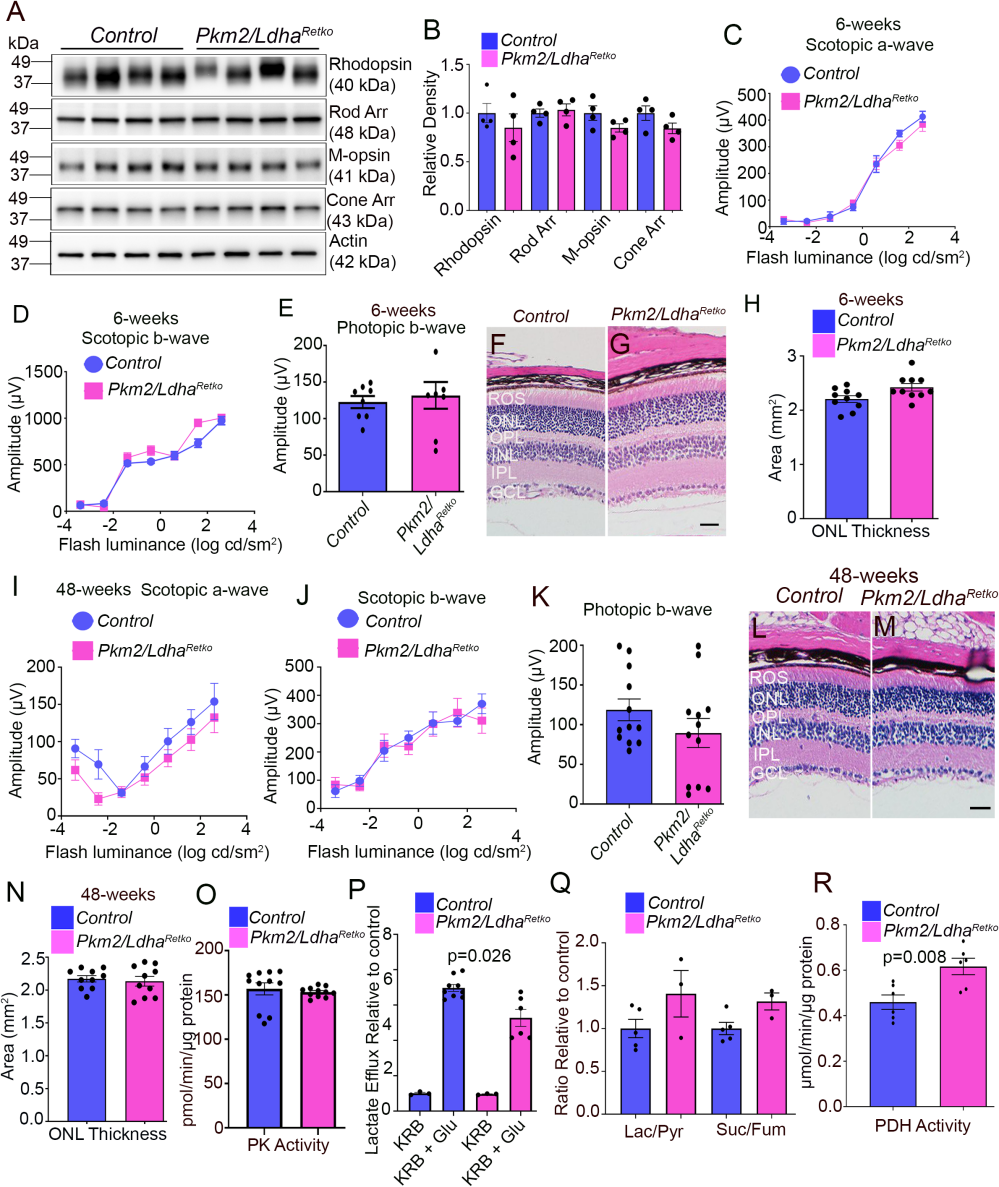
Structural, biochemical, and functional characterization of the effects of PKM2 and LDHA loss in the retina. Retinal lysates from 48 week old control and *Pkm2/Ldha* ^*Retko*^ mice were immunoblotted with antibodies against rhodopsin, rod arrestin, M‐opsin, and cone arrestin (A), and the levels were normalized to Actin (B). Data are mean ± SEM (*n* = *4*). Scotopic a‐wave (C), scotopic b‐wave (D), and photopic b‐wave (E) amplitudes were measured in 6 week old control and *Pkm2Ldha* ^*Retko*^ mice. Data are mean ± SEM (*n* = *8*). Six week old control and *Pkm2Ldha* ^*Retko*^ mouse retinas were stained with hematoxylin and eosin and examined for morphology (F, G). Scale bar = 50 μm. Quantification of ONL thickness was performed using NIH ImageJ software (H). Data are mean ± SEM (*n* = *10*). Scotopic a‐wave (I), scotopic b‐wave (J), and photopic b‐wave (K) amplitudes were measured in 48 week old control and *Pkm2Ldha* ^*Retko*^ mice. Data are mean ± SEM (*n* = *12*). Forty‐eight week old control and *Pkm2Ldha* ^*Retko*^ mouse retina sections were stained with hematoxylin and eosin and examined for morphology (L, M). Scale bar = 50 μm. Quantification of ONL thickness was performed using NIH ImageJ software (N). Data are mean ± SEM (*n* = *10*). Pyruvate kinase activity was measured in retinas from control and *Pkm2/Ldha* ^*Retko*^ mice (O). Data are mean ± SEM (*n* = *12*). Ex vivo retinal explants from 48 week old control and *Pkm2/Ldha* ^*Retko*^ mice were incubated in Krebs–Ringer–Bicarbonate (KRB) buffer containing 5 mM glucose, and lactate release was quantified after 30 min (P). Data are mean ± SEM (*n* = *6*). Lactate/pyruvate and succinate/fumarate ratios were determined from control and *Pkm2/Ldha* ^*Retko*^ mice (Q). Data are mean ± SEM (*n* = *5*). Pyruvate dehydrogenase (PDH) activity was measured in control and *Pkm2/Ldha* ^*Retko*^ *retinas* (R). Data are mean ± SEM (*n* = *6*).

Forty‐eight week old control and *Pkm2/Ldha* ^*Retko*^ mouse retinas were immunoblotted with antibodies against rhodopsin, rod arrestin, M‐opsin, and cone arrestin, and the band intensities were normalized to actin. The results show no significant differences in protein levels between control and *Pkm2/Ldha* ^*Retko*^ retinas (Figure 6A,B). Retinal sections stained with the Müller cell markers GS and GFAP showed no change in GS expression; however, GFAP expression was increased in *Pkm2/Ldha* ^*Retko*^ mice compared to control retinas (Figure S4A–F).

### 3.9 Age‐Dependent Alterations in Retinal Structure and Function Following Pan‐Retinal Deletion of PKM2 and LDHA

Functional evaluation of six week old control and *Pkm2/Ldha* ^*Retko*^ mice shows that no significant differences were observed in scotopic a‐wave, scotopic b‐wave, or photopic b‐wave amplitudes between the two groups (Figure 6C–E). Histological analysis further confirmed the absence of structural changes in *Pkm2/Ldha* ^*Retko*^ retinas compared with control retinas (Figure 6F–H). Functional evaluation of 48 week old mice demonstrated no significant differences in scotopic a‐wave, scotopic b‐wave, or photopic b‐wave amplitudes between control and *Pkm2/Ldha* ^*Retko*^ mice (Figure 6I–K). Histological analysis further confirmed the absence of structural changes in *Pkm2/Ldha* ^*Retko*^ retinas compared with control retinas (Figure 6L–N).

### 3.10 Biochemical Characterization of Mice Lacking PKM2 and LDHA in the Retina

We measured pyruvate kinase activity in *Pkm2/Ldha* ^*Retko*^ mouse retinas. We found no significant difference compared to control retinas (Figure 6O). In contrast, we found reduced pyruvate kinase activity in *Ldha* ^*Rodko*^ and significantly reduced activity in *Pkm2/Ldha* ^*Rodko*^ mouse retinas compared to control retinas (Figure S5A,B). Retinas from control and *Pkm2/Ldha* ^*Retko*^ mice were incubated with glucose, and lactate release was measured. Lactate efflux was significantly lower in *Pkm2/Ldha* ^*Retko*^ retinas compared to controls (Figure 6P). Interestingly, there was no reduction in the lactate efflux in *Ldha* ^*Rodko*^ and *Pkm2/Ldha* ^*Rodko*^ mouse retinas compared to control retinas (Figure S5C,D). These observations suggest that lactate may be produced by other retinal cell types. We carried out the lactate efflux assay with control and *Ldha* ^*Rodko*^ mouse retinas in the presence of glucose and glucose plus UK5050, a potent inhibitor of the mitochondrial pyruvate carrier (MPC), which blocks the entry of pyruvate into the mitochondria [17]. We found a significant reduction in the lactate release in *Ldha* ^*Retko*^ mouse retinas treated with the MPC inhibitor, but not in control retinas (Figure S5E). These observations suggest that mitochondria may produce lactate. To determine whether mitochondria express LDH isoforms, we isolated mitochondria and cytosol from the C57Bl6 mouse retina. Immunoblot analysis using the mitochondrial marker antibody, voltage‐dependent anion channel (VDAC), confirmed that VDAC is present only in mitochondrial preparation, not in the cytosol (Figure S5F). The mitochondrial fraction shows enrichment for LDHB and, to a lesser extent, LDHA relative to the cytosol (Figure S5F,G). To determine whether mitochondria can convert pyruvate to lactate, isolated mitochondria were incubated with buffer alone, glucose, phosphoenolpyruvate (PEP), or pyruvate (PYR). Control samples contained buffer with the same additives. Lactate efflux was then measured. Lactate release was observed only in the presence of pyruvate, not with glucose or PEP (Figure S5H), suggesting that mitochondria may contribute to lactate generation under these conditions.

**FIGURE 7:**
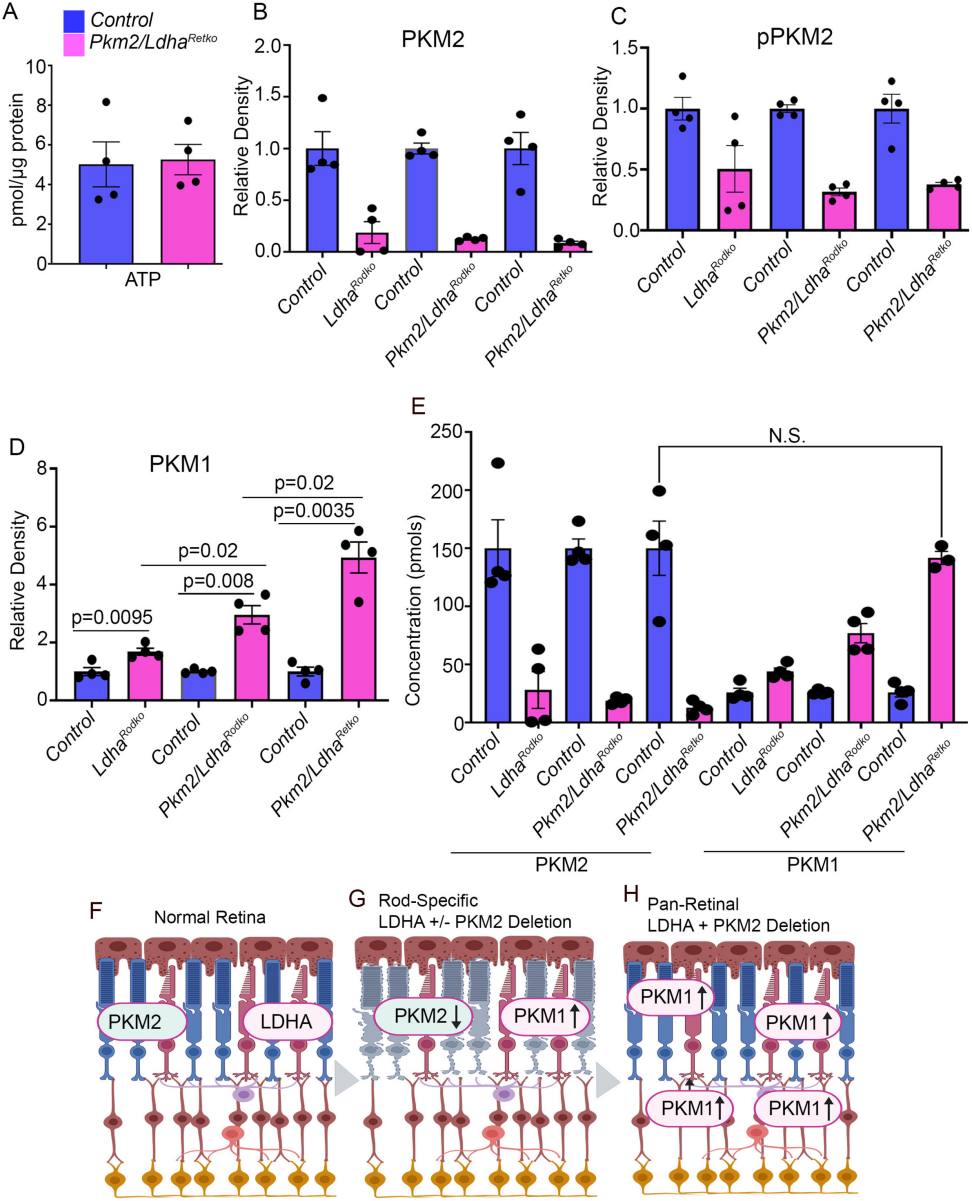
Effects of combined PKM2 and LDHA loss in the retina on retinal metabolism. ATP levels were assessed in control and *Pkm2/Ldha* ^*Retko*^ *retinas* (A). Data are mean ± SEM (*n* = *4*). Relative levels of PKM2 (B), pPKM2 (C), PKM1 (D), and the molar concentrations of PKM2 and PKM1 were measured in control, *Ldha* ^*Rodko*^, *Pkm2/Ldha* ^*Rodko*^, and *Pkm2/Ldha* ^*Retko*^ mice (E). Data are mean ± SEM (*n* = *4*). Loss of either PKM2 or LDHA induces compensatory upregulation of PKM1, introducing PKM1 as a key determinant of metabolic adaptation in the retina. In the normal retina, PKM2 and LDHA are maintained at defined levels that support photoreceptor metabolism (F). Rod‐specific deletion of LDHA, either alone or in combination with PKM2, results in age‐dependent structural decline and retinal degeneration because PKM1 induction does not reach levels comparable to PKM2 (G). In contrast, pan‐retinal deletion of both LDHA and PKM2 increases PKM1 expression to levels similar to PKM2 (H), restores glycolytic flux, and preserves retinal structure and function. Together, these findings support a PKM isoform balance threshold model in which retinal survival depends on sufficient PKM1 compensation following PKM2 loss. When PKM1 induction is inadequate, degeneration ensues; when PKM1 reaches levels comparable to PKM2, retinal integrity is maintained.

The lactate/pyruvate and succinate/fumarate ratios were higher in *Pkm2/Ldha* ^*Retko*^ retinas; however, the differences were not statistically significant compared to control retinas (Figure 6Q). Pyruvate dehydrogenase (PDH) activity was significantly increased in *Pkm2/Ldha* ^*Retko*^ retinas compared to control retinas (Figure 6R). However, ATP levels were unchanged between the two groups (Figure 7A).

We examined how loss of LDHA in rods, loss of LDHA and PKM2 in rods, and loss of LDHA and PKM2 in the entire retina affect PKM1 expression. Deleting LDHA in rods resulted in more than an 80% reduction in PKM2, and a similar decrease was observed in both *Pkm2/Ldha* ^*Rodko*^ and *Pkm2/Ldha* ^*Retko*^ mice (Figure 7B). Corresponding to the reduction in PKM2, PKM2 phosphorylation was also decreased across all three genotypes, with no significant differences between them (Figure 7C). In contrast, PKM1 levels gradually increased across groups, with significant differences observed between control and *Ldha* ^*Rodko*^, control and *Pkm2/Ldha* ^*Rodko*^, control and *Pkm2/Ldha* ^*Retko*^, and among the three knockout genotypes themselves (Figure 7D).

In the retina, PKM2 is present at ~150 pmol, whereas PKM1 is present at ~26 pmol [18, 19]. We converted the levels of PKM2 and PKM1 in *Ldha* ^*Rodko*^, *Pkm2/Ldha* ^*Rodko*^ and *Pkm2/Ldha* ^*Retko*^ mice into their respective molar concentrations (Figure 7E). Taking PKM2 concentration as 150 pmol and PKM1 concentration as 26 pmols, loss of *Ldha* in rods results in the increase of 26 pmol of PKM1 to 44 pmol, which is not comparable to the concentration of PKM2 in the retina (Figure 7E). Combined loss of PKM2 and LDHA in rods increased levels of PKM1 to 77 pmol, significantly higher than control (26 pmol) and *Ldha* ^*Rodko*^ (44 pmol), still lower than the concentration of PKM2 (150 pmol) (Figure 7E). Interestingly, loss of PKM2 and LDHA in the retina, significantly elevated the levels of PKM1 to 142 pmols, which is considerably higher than *Ldha* ^*Rodko*^ and *Pkm2/Ldha* ^*Rodko*^ mouse retina and the levels are comparable to the levels of PKM2 in the retina (Figure 7E).

## 4 Discussion

Both PKM2 and LDHA are key enzymes implicated in aerobic glycolysis, particularly in the context of cancer metabolism [8, 20]. PKM2 and LDHA function together to sustain the high glycolytic flux needed for cell proliferation. The low activity of dimeric PKM2 facilitates the generation of essential metabolic intermediates, whereas the high activity of LDHA converts pyruvate to lactate and regenerates key cofactors. This coordination enables glycolysis to run efficiently and rapidly, supporting the cell's anabolic needs.

Although aerobic glycolysis is typically linked to proliferating tumor cells, postmitotic photoreceptors also rely on this metabolic program. Each day, the RPE removes about 10% of the photoreceptor tips, creating a continuous need for new lipid and protein synthesis to maintain photoreceptor structure [1, 21]. This persistent anabolic demand resembles the metabolic requirements of dividing cells and explains the prominent expression of LDHA in photoreceptors. Current understanding indicates that, in tumors, aerobic glycolysis arises when mitochondrial shuttle systems, such as the malate–aspartate and glycerol‐3‐phosphate shuttles, reach their limits in oxidizing cytosolic NADH [9]. When these pathways are saturated, LDHA becomes essential for regenerating NAD^+^, allowing glycolysis to continue. In photoreceptors, high glycolytic activity combined with daily renewal demands likely pushes these shuttles to capacity, making LDHA‐mediated lactate production necessary. PKM2 supports this process by directing glycolytic intermediates into biosynthetic pathways. Thus, we focused our study on LDHA and its functional relationship with PKM2 to understand how these enzymes support the metabolic requirements for sustaining photoreceptor integrity and health.

The importance of this study lies in the finding that loss of LDHA, and the combined loss of LDHA and PKM2, in photoreceptor cells causes progressive age‐related retinal degeneration. In contrast, the combined loss of LDHA and PKM2 throughout the retina is associated with resistance to photoreceptor degeneration. The novel observation in this study is that loss of LDHA in rods leads to downregulation of PKM2, thereby upregulating PKM1. The upregulation of PKM1 in rods lacking PKM2 and LDHA is significantly higher than in rods lacking LDHA. PKM2 is known to suppress the expression of PKM1 [22]; however, loss of LDHA suppresses PKM2, leading to PKM upregulation, suggesting that LDHA may regulate PKM2 transcription. In the retina, the molar concentration of PKM2 is ~5‐fold higher than PKM1 [18, 19]. The upregulation of PKM1 in the absence of LDHA, or in the combined absence of LDHA and PKM2, in rods did not reach levels comparable to those of PKM2, which could explain the degeneration phenotype observed in both mouse models. Interestingly, the combined loss of LDHA and PKM2 in all retinal neurons increased PKM1 to levels comparable to PKM2, which may contribute to enhanced retinal resilience and preservation of structure and function.

PKM1 is expressed in terminally differentiated cells [22, 23], whereas PKM2 is present in many cell types, especially those with high metabolic activity, including mitotic tumor cells, proliferating endothelial cells, and developing fetal tissues [3]. The function of PKM1 in tumor progression remains debated. Recent work demonstrates that PKM1 can enhance the growth of several tumor cell lines by promoting glucose catabolism without affecting biosynthetic pathways [24]. PKM1‐driven tumor progression is linked to its ability to activate autophagy and mitophagy, and in this context, PKM1—but not PKM2—supports malignant cell proliferation [24]. Retinal cell survival in mice lacking LDHA and PKM2 is associated with increased PKM1 expression and may reflect a contributory role of PKM1, similar to observations reported in tumor progression. The retina is a heterogeneous tissue composed of several neuronal cells [25]. Our TRAP analysis shows the expression levels of LDHA and LDHB in rods, cones, RPE, Müller cells, and RGCs. The expression levels of PKM1, PKM2, LDHA, and LDHB in other retinal neurons are currently unknown. The Chx10‐Cre we used in this study deletes PKM2 and LDHA in all retinal progenitor cells during retinal development. The upregulation of PKM1 in retinal cells lacking LDHA and PKM2 could be coming from other retinal cells as well. This idea is further supported by the observation that deleting either LDHA or LDHA and PKM2 in rods did not affect lactate efflux, whereas deleting both LDHA and PKM2 in all retinal cells resulted in reduced lactate efflux, indicating that other retinal cells contribute to lactate release in response to glucose. Further studies are needed to identify the expression levels of these enzymes in all retinal cell types.

In isolated retinal mitochondria, lactate production was detected only in the presence of pyruvate, and not with glucose or PEP (Figure S5H), suggesting that mitochondria may generate lactate under specific substrate conditions. Consistent with this observation, studies in cancer cells have reported that lactate metabolism is associated with mammalian mitochondria and shown, by transmission electron microscopy, that LDHB localizes to mitochondria [12]. Together, these findings suggest a possible link between lactate metabolism and mitochondrial function, although further studies are needed to clarify this relationship.

Despite changes in pyruvate kinase activity, PDH regulation, and lactate flux, total ATP levels remain stable, suggesting the presence of compensatory mechanisms after PKM2 loss (*Pkm2/Ldha* ^*Retko*^). Increased PKM1 expression may facilitate redistribution of glycolytic intermediates toward mitochondrial oxidation, potentially supporting ATP production through oxidative phosphorylation. Whole‐retina deletion may also permit metabolic buffering among retinal cell types, allowing substrate and redox exchange across the tissue. Because ATP concentration reflects a tightly regulated steady‐state pool rather than metabolic flux, these mechanisms could help maintain ATP levels despite altered glycolytic dynamics. The preservation of photoreceptors following whole‐retina deletion supports the concept of metabolic cooperation among retinal cell types. In the context of PKM2 loss, increased PKM1 expression across the retina may allow neuron–glia or inter‐neuronal metabolic buffering, facilitating redistribution of glycolytic activity and metabolic intermediates. This tissue‐level compensation likely sustains energy balance and structural integrity, whereas cell‐restricted disruption limits compensatory capacity and promotes degeneration. Further studies are required to define the mechanisms underlying this cooperative metabolic response.

In summary, our study demonstrates that LDHA and PKM2 are essential for sustaining photoreceptor metabolic demands and maintaining retinal integrity. Loss of LDHA, or combined loss of LDHA and PKM2 in rods, leads to age‐related degeneration, accompanied by reduced PKM2 and incomplete compensatory upregulation of PKM1. In contrast, deleting both enzymes across all retinal neurons induces a robust increase in PKM1 and protects against degeneration. We propose a PKM isoform balance threshold model in which retinal survival depends on the level of PKM1 compensation after PKM2 loss. Reduced PKM1 expression leads to age‐dependent retinal degeneration, whereas increasing PKM1 to levels comparable to PKM2 permits cell type–dependent metabolic compensation. In this setting, PKM1 supports glycolytic flux and helps maintain retinal structure and function (Figure 7F). These findings highlight cell‐type‐specific metabolic compensation and its importance for preserving retinal health.

## Author Contributions

R.V.S.R. conceived and designed the study. R.V.S.R., A.R., T.M.B., and L.J.T. conducted the experiments. R.V.S.R. and A.R. performed data analysis. R.V.S.R. and A.R. interpreted the results. R.V.S.R. drafted and wrote the manuscript, incorporating feedback and critical input from all co‐authors. All authors reviewed and approved the final version of the manuscript.

## Funding

Grants from the National Institutes of Health EY035282, NEI Core grant EY021725, Presbyterian Health Foundation, and an unrestricted grant from Research to Prevent Blindness Inc. awarded to the OUHSC Department of Ophthalmology.

## Conflicts of Interest

The authors declare no conflicts of interest.

## Supporting information


**Figure S1:** Expression of PKM1 in the *Ldha* ^*Rodko*^ mouse retina. Retinal sections from control (A) and *Ldha* ^*Rodko*^ (B) mice were immunostained with antibody against PKM1. Scale bar = 50 μm. These images correspond to those shown in Figure 1, panels P and Q, and were re‐stained and captured at lower saturation.
**Figure S2:** Expression of PKM2, LDHA, and PKM1 in the *Pkm2/Ldha^Rodko^* mouse retina. Retinal sections from control (A–C) and *Pkm2/Ldha^Rodko^* (D–F) mice were immunostained with antibodies against rhodopsin and PKM2 (A, D), rhodopsin and LDHA (B, E), and rhodopsin and PKM1 (C, F). Scale bar = 50 μm.
**Figure S3:** Expression of LDHB and phosphorylation status of PKM2 in the *Pkm2/Ldha^Rodko^* mouse retina. Retinal sections from control (A, C) and *Pkm2/Ldha^Rodko^* (B, D) mice were immunostained with antibodies against rhodopsin and LDHB (A, B) or rhodopsin and phosphorylated PKM2 (pPKM2) (C, D). Scale bar = 50 μm.
**Figure S4:** Expression of GS and GFAP in control and *Pkm2/Ldha^Retko^* mice. Control (A‐C) and *Pkm2/Ldha^Retko^* (D‐F) mouse retina sections were immunostained with GFAP (A, D) and GS (B‐E) antibodies. Panels C and F represent the merged image of GS and GFAP. Scale bar = 50 μm.
**Figure S5:** Effect of loss of PKM2 and LDHA in the retina on pyruvate kinase activity and lactate release. Pyruvate kinase activity was measured in retinas from *Ldha^Retko^* (A) and *Pkm2/Ldha^Rodko^* (B) mice. Data are mean ± SEM (*n* = 6). Ex vivo retinal explants from *Ldha^Rodko^* (C) and *Pkkm2/Ldha^Rodko^* (D) mice were incubated in KRB buffer containing 5 mM glucose, and lactate release was quantified after 30 min. Data are mean ± SEM (*n* = 6). Lactate efflux assay from ex vivo control and *Ldha^Rodko^* mice in the presence of glucose and glucose plus MPC inhibitor UK5050, and lactate release was quantified after 30 min (E). Data are mean ± SEM (*n* = 6). Cytosol and mitochondria were prepared from C57Bl6 mice, and the fractions were immunoblotted with antibodies against VDAC, LDHA, and LDHB (F). The levels of LDHA and LDHB in the mitochondria are normalized to cytosolic LDHA and LDHB (G). The lactate efflux assay was carried out using mitochondrial preparation in the presence of glucose, phosphoenolpyruvate (PEP), and pyruvate (PEP), and control experiments were conducted with buffer instead of mitochondria (H). Data are mean ± SEM (*n* = 3).
**Table S1:** Real‐time PCR primers to quantify the expression of Ldha and Ldhb in retina, rod photoreceptor, Cone, Müller, RGC, and RPE cells.
**Table S2:** Antibodies used for immunofluorescence (IF) and immunoblot Analysis.

## Data Availability

All data are included in this article and/or Supporting Information.

## References

[1] C. Punzo , W. Xiong , and C. L. Cepko , “Loss of Daylight Vision in Retinal Degeneration: Are Oxidative Stress and Metabolic Dysregulation to Blame?,” Journal of Biological Chemistry 287 (2012): 1642–1648. 22074929 10.1074/jbc.R111.304428 PMC3265845

[2] J. B. Hurley , K. J. Lindsay , and J. Du , “Glucose, Lactate, and Shuttling of Metabolites in Vertebrate Retinas,” Journal of Neuroscience Research 93 (2015): 1079–1092. 25801286 10.1002/jnr.23583 PMC4720126

[3] R. V. S. Rajala , “Aerobic Glycolysis in the Retina: Functional Roles of Pyruvate Kinase Isoforms,” Frontiers in Cell and Developmental Biology 8 (2020): 266. 32426353 10.3389/fcell.2020.00266 PMC7203425

[4] Y. Chinchore , T. Begaj , D. Wu , E. Drokhlyansky , and C. L. Cepko , “Glycolytic Reliance Promotes Anabolism in Photoreceptors,” eLife 6 (2017): e25946. 28598329 10.7554/eLife.25946 PMC5499945

[5] T. J. Wubben , M. Pawar , A. Smith , K. Toolan , H. Hager , and C. G. Besirli , “Photoreceptor Metabolic Reprogramming Provides Survival Advantage in Acute Stress While Causing Chronic Degeneration,” Scientific Reports 7 (2017): 17863. 29259242 10.1038/s41598-017-18098-z PMC5736549

[6] O. Warburg , “On Respiratory Impairment in Cancer Cells,” Science 124 (1956): 269–270. 13351639

[7] O. Warburg , “On the Origin of Cancer Cells,” Science 123 (1956): 309–314. 13298683 10.1126/science.123.3191.309

[8] H. R. Christofk , M. G. Vander Heiden , M. H. Harris , et al., “The M2 Splice Isoform of Pyruvate Kinase Is Important for Cancer Metabolism and Tumour Growth,” Nature 452 (2008): 230–233. 18337823 10.1038/nature06734

[9] Y. Wang , E. Stancliffe , R. Fowle‐Grider , et al., “Saturation of the Mitochondrial NADH Shuttles Drives Aerobic Glycolysis in Proliferating Cells,” Molecular Cell 82 (2022): 3270–3283.e3279. 35973426 10.1016/j.molcel.2022.07.007 PMC10134440

[10] R. V. Rajala , A. Rajala , C. Kooker , Y. Wang , and R. E. Anderson , “The Warburg Effect Mediator Pyruvate Kinase M2 Expression and Regulation in the Retina,” Scientific Reports 6 (2016): 37727. 27883057 10.1038/srep37727 PMC5121888

[11] J. Y. S. Han , J. Kinoshita , S. Bisetto , et al., “Role of Monocarboxylate Transporters in Regulating Metabolic Homeostasis in the Outer Retina: Insight Gained From Cell‐Specific Bsg Deletion,” FASEB Journal 34 (2020): 5401–5419. 32112484 10.1096/fj.201902961R PMC7849204

[12] Y. J. Chen , N. G. Mahieu , X. Huang , et al., “Lactate Metabolism Is Associated With Mammalian Mitochondria,” Nature Chemical Biology 12 (2016): 937–943. 27618187 10.1038/nchembio.2172 PMC5069139

[13] A. Rajala , R. Rajala , G. K. Gopinadhan Nair , and R. V. S. Rajala , “Atlas of Phosphoinositide Signatures in the Retina Identifies Heterogeneity Between Cell Types,” PNAS Nexus 2 (2023): pgad063. 37007713 10.1093/pnasnexus/pgad063 PMC10062291

[14] A. Rajala , M. A. Bhat , K. Teel , G. K. Gopinadhan Nair , L. Purcell , and R. V. S. Rajala , “The Function of Lactate Dehydrogenase A in Retinal Neurons: Implications to Retinal Degenerative Diseases,” PNAS Nexus 2 (2023): pgad038. 36896135 10.1093/pnasnexus/pgad038 PMC9991461

[15] G. Sutendra , P. Dromparis , A. Kinnaird , et al., “Mitochondrial Activation by Inhibition of PDKII Suppresses HIF1a Signaling and Angiogenesis in Cancer,” Oncogene 32 (2013): 1638–1650. 22614004 10.1038/onc.2012.198

[16] M. S. Patel , N. S. Nemeria , W. Furey , and F. Jordan , “The Pyruvate Dehydrogenase Complexes: Structure‐Based Function and Regulation,” Journal of Biological Chemistry 289 (2014): 16615–16623. 24798336 10.1074/jbc.R114.563148 PMC4059105

[17] N. M. Vacanti , A. S. Divakaruni , C. R. Green , et al., “Regulation of Substrate Utilization by the Mitochondrial Pyruvate Carrier,” Molecular Cell 56 (2014): 425–435. 25458843 10.1016/j.molcel.2014.09.024 PMC4267523

[18] K. J. Lindsay , J. Du , S. R. Sloat , et al., “Pyruvate Kinase and Aspartate‐Glutamate Carrier Distributions Reveal Key Metabolic Links Between Neurons and Glia in Retina,” Proceedings of the National Academy of Sciences of the United States of America 111 (2014): 15579–15584. 25313047 10.1073/pnas.1412441111 PMC4217417

[19] A. Rajala , Y. Wang , R. S. Brush , et al., “Pyruvate Kinase M2 Regulates Photoreceptor Structure, Function, and Viability,” Cell Death & Disease 9 (2018): 240. 29445082 10.1038/s41419-018-0296-4 PMC5833680

[20] Y. Feng , Y. Xiong , T. Qiao , X. Li , L. Jia , and Y. Han , “Lactate Dehydrogenase A: A Key Player in Carcinogenesis and Potential Target in Cancer Therapy,” Cancer Medicine 7 (2018): 6124–6136. 30403008 10.1002/cam4.1820 PMC6308051

[21] M. M. LaVail , “Rod Outer Segment Disk Shedding in Rat Retina: Relationship to Cyclic Lighting,” Science 194 (1976): 1071–1074. 982063 10.1126/science.982063

[22] W. J. Israelsen and M. G. Vander Heiden , “Pyruvate Kinase: Function, Regulation and Role in Cancer,” Seminars in Cell & Developmental Biology 43 (2015): 43–51. 26277545 10.1016/j.semcdb.2015.08.004 PMC4662905

[23] S. Amin , P. Yang , and Z. Li , “Pyruvate Kinase M2: A Multifarious Enzyme in Non‐Canonical Localization to Promote Cancer Progression,” Biochimica et Biophysica Acta. Reviews on Cancer 1871 (2019): 331–341. 30826427 10.1016/j.bbcan.2019.02.003

[24] M. Morita , T. Sato , M. Nomura , et al., “PKM1 Confers Metabolic Advantages and Promotes Cell‐Autonomous Tumor Cell Growth,” Cancer Cell 33 (2018): 355–367.e357. 29533781 10.1016/j.ccell.2018.02.004

[25] M. Hoon , H. Okawa , L. Della Santina , and R. O. Wong , “Functional Architecture of the Retina: Development and Disease,” Progress in Retinal and Eye Research 42 (2014): 44–84. 24984227 10.1016/j.preteyeres.2014.06.003 PMC4134977

