# Supplementary material for: Cell‐Type‐Dependent Metabolic Compensation Preserves Photoreceptor Survival Through Pyruvate Kinase Isoform Balance
Source: FASEB J. 2026 Mar 31;40(7):e71730. doi: 10.1096/fj.202505064R (PMC13037751; doi:10.1096/fj.202505064R)
Supplement: Supplementary file 1 — Figure S1: Expression of PKM1 in the Ldha Rodko mouse retina. Retinal sections from control (A) and Ldha Rodko (B) mice were immunostained with antibody against PKM1. Scale bar = 50 μm. These images correspond to those shown in Figure 1, panels P and Q, and were re‐stained and captured at lower saturation. Figure S2: Expression of PKM2, LDHA, and PKM1 in the Pkm2/LdhaRodko mouse retina. Retinal sections from control (A–C) and Pkm2/LdhaRodko (D–F) mice were immunostained with antibodies against rhodopsin and PKM2 (A, D), rhodopsin and LDHA (B, E), and rhodopsin and PKM1 (C, F). Scale bar = 50 μm. Figure S3: Expression of LDHB and phosphorylation status of PKM2 in the Pkm2/LdhaRodko mouse retina. Retinal sections from control (A, C) and Pkm2/LdhaRodko (B, D) mice were immunostained with antibodies against rhodopsin and LDHB (A, B) or rhodopsin and phosphorylated PKM2 (pPKM2) (C, D). Scale bar = 50 μm. Figure S4: Expression of GS and GFAP in control and Pkm2/LdhaRetko mice. Control (A‐C) and Pkm2/LdhaRetko (D‐F) mouse retina sections were immunostained with GFAP (A, D) and GS (B‐E) antibodies. Panels C and F represent the merged image of GS and GFAP. Scale bar = 50 μm. Figure S5: Effect of loss of PKM2 and LDHA in the retina on pyruvate kinase activity and lactate release. Pyruvate kinase activity was measured in retinas from LdhaRetko (A) and Pkm2/LdhaRodko (B) mice. Data are mean ± SEM (n = 6). Ex vivo retinal explants from LdhaRodko (C) and Pkkm2/LdhaRodko (D) mice were incubated in KRB buffer containing 5 mM glucose, and lactate release was quantified after 30 min. Data are mean ± SEM (n = 6). Lactate efflux assay from ex vivo control and LdhaRodko mice in the presence of glucose and glucose plus MPC inhibitor UK5050, and lactate release was quantified after 30 min (E). Data are mean ± SEM (n = 6). Cytosol and mitochondria were prepared from C57Bl6 mice, and the fractions were immunoblotted with antibodies against VDAC, LDHA, and LDHB (F). The levels of LDHA and LD [file FSB2-40-e71730-s001.docx]

**
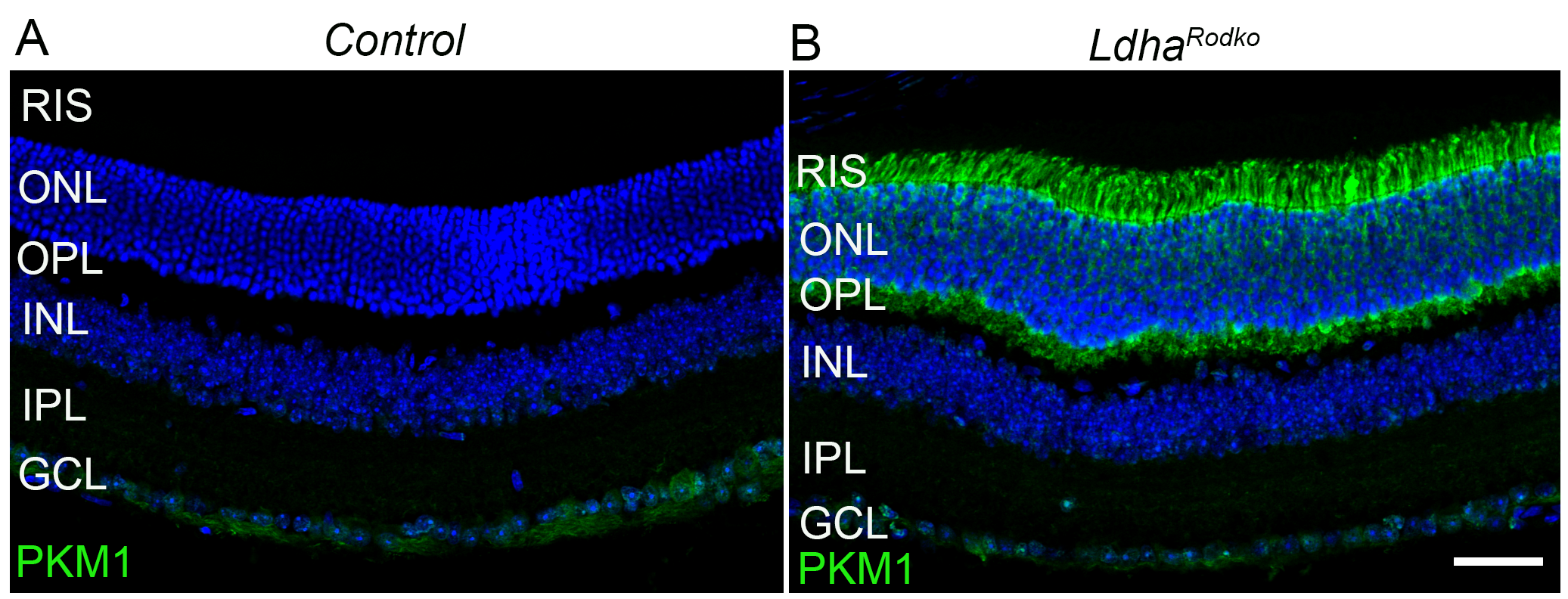
**

**Figure S1. Expression of PKM1 in the *Ldha^Rodko^* mouse retina.** Retinal sections from control (A) and *Ldha^Rodko^* (B) mice were immunostained with antibody against PKM1. Scale bar = 50 μm. These images correspond to those shown in Figure 1, panels P and Q, and were re-stained and captured at lower saturation.


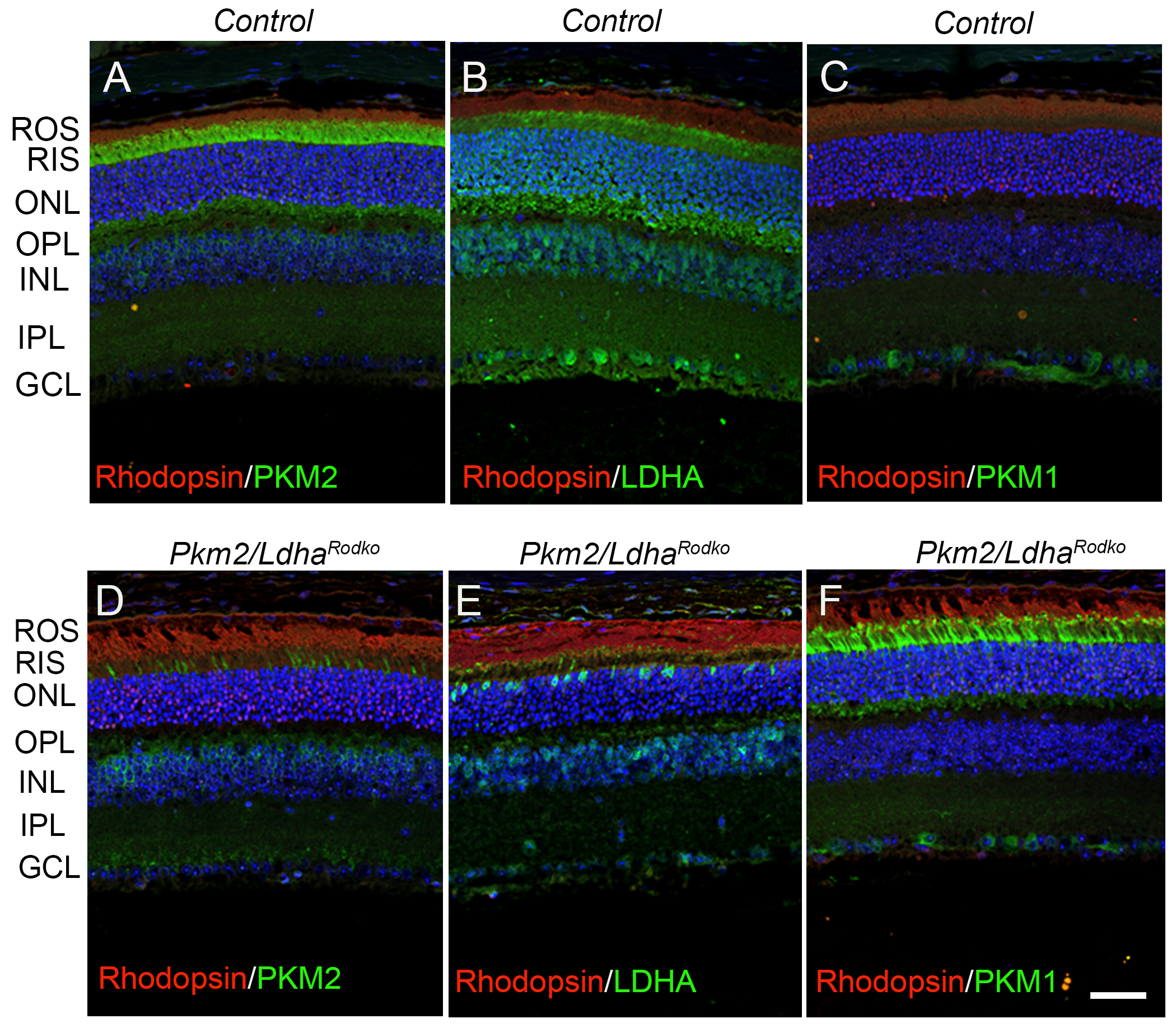
**Figure S2. Expression of PKM2, LDHA, and PKM1 in the *Pkm2/Ldha^Rodko^* mouse retina.** Retinal sections from control (A–C) and *Pkm2/Ldha^Rodko^* (D–F) mice were immunostained with antibodies against rhodopsin and PKM2 (A, D), rhodopsin and LDHA (B, E), and rhodopsin and PKM1 (C, F). Scale bar = 50 μm.

**Figure S3.**
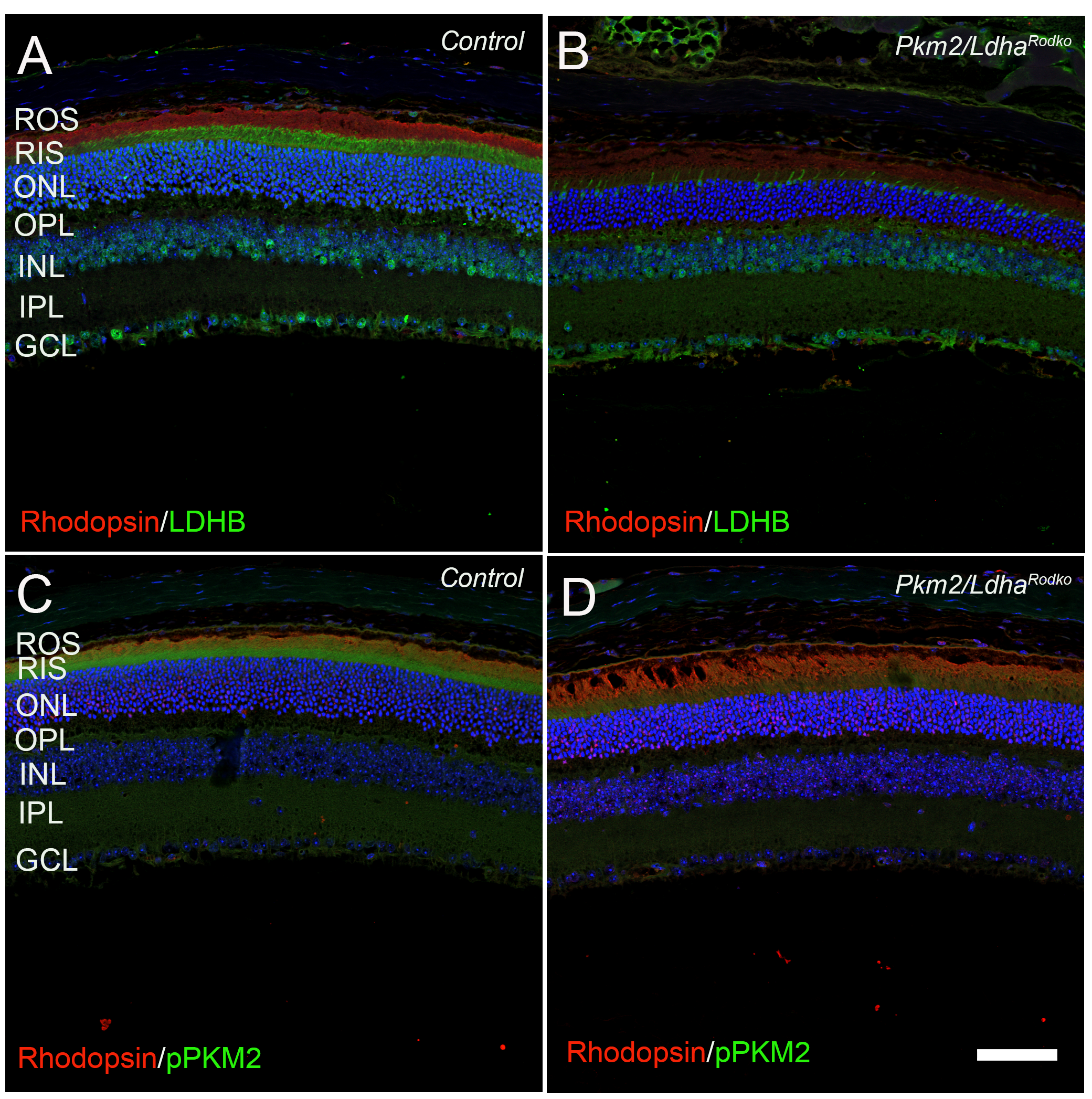
**Expression of LDHB and phosphorylation status of PKM2 in the *Pkm2/Ldha^Rodko^* mouse retina.** Retinal sections from control (A, C) and *Pkm2/Ldha^Rodko^* (B, D) mice were immunostained with antibodies against rhodopsin and LDHB (A, B) or rhodopsin and phosphorylated PKM2 (pPKM2) (C, D). Scale bar = 50 μm.


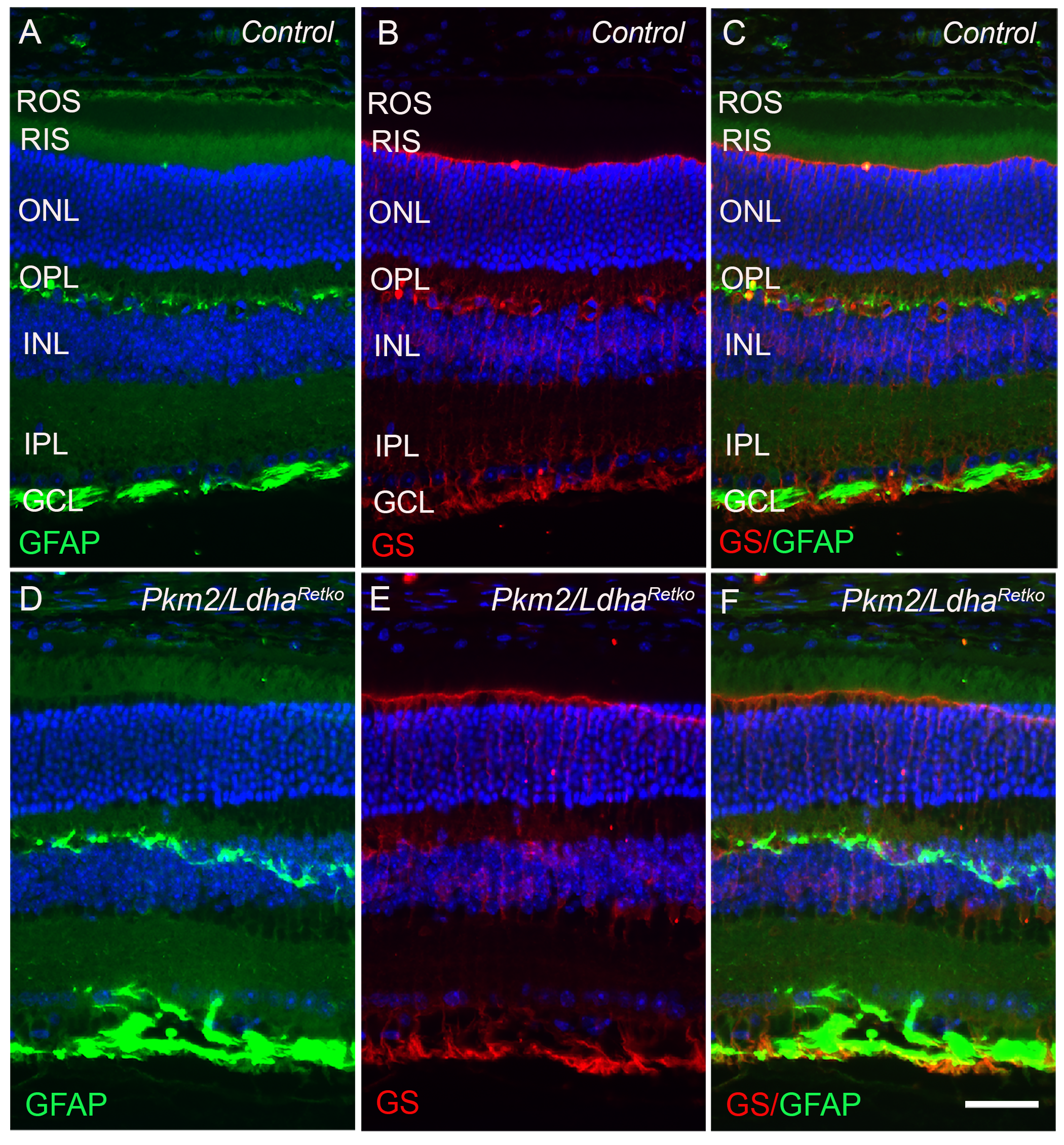
**Figure S4. Expression of GS and GFAP in control and *Pkm2/Ldha^Retko^* mice.** Control (A-C) and *Pkm2/Ldha^Retko^* (D-F) mouse retina sections were immunostained with GFAP (A, D) and GS (B-E) antibodies. Panels C and F represent the merged image of GS and GFAP. Scale bar = 50 µm.


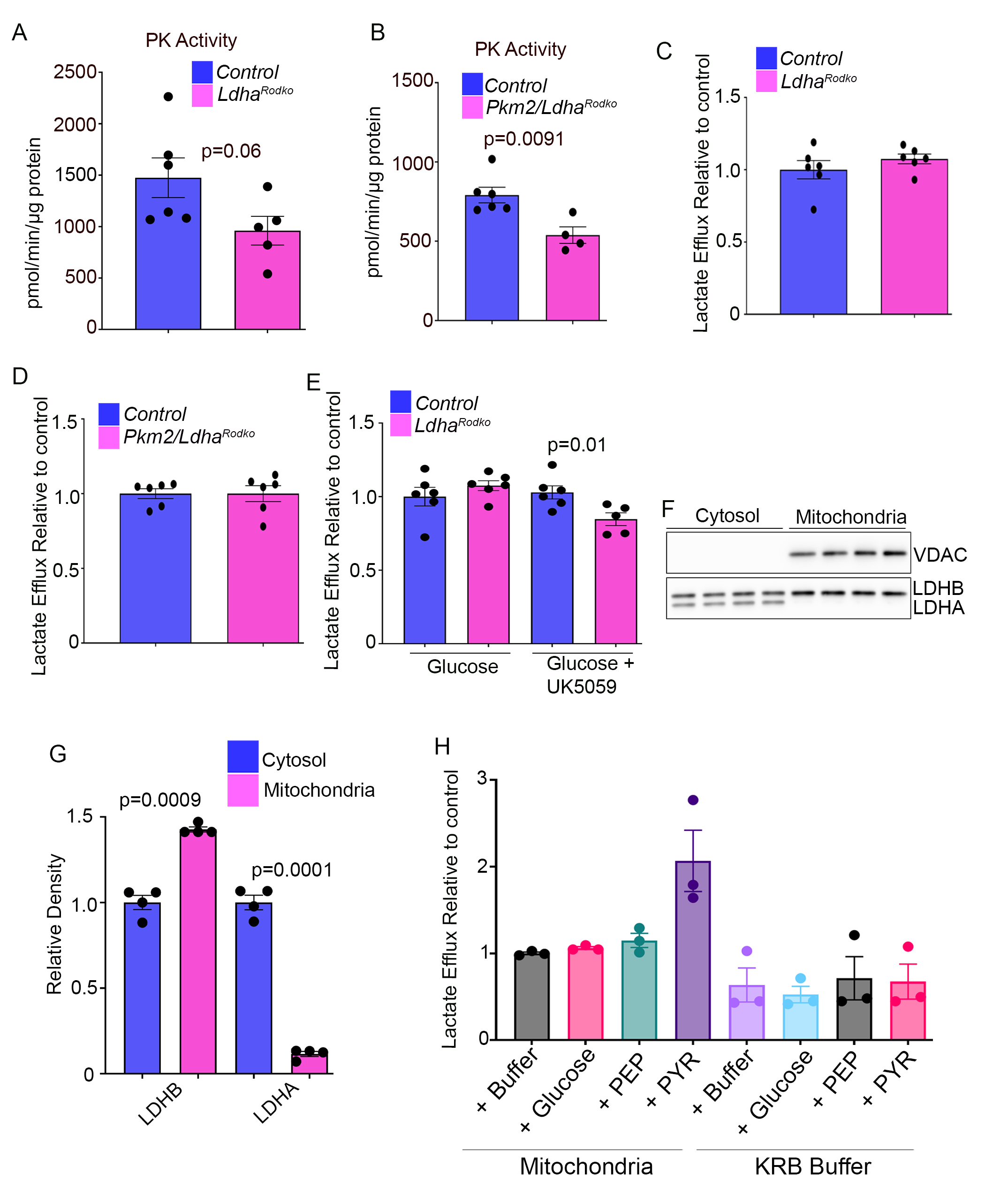
**Figure S5. Effect of loss of PKM2 and LDHA in the retina on pyruvate kinase activity and lactate release.** Pyruvate kinase activity was measured in retinas from *Ldha^Retko^* (A) and *Pkm2/Ldha^Rodko^* (B) mice. Data are mean ± *SEM (n=6).* *Ex vivo* retinal explants from *Ldha^Rodko^* (C) and *Pkkm2/Ldha^Rodko^* (D) mice were incubated in KRB buffer containing 5 mM glucose, and lactate release was quantified after 30 min. Data are mean ± *SEM (n=6).* Lactate efflux assay from *ex vivo* control and *Ldha^Rodko^* mice in the presence of glucose and glucose plus MPC inhibitor UK5050, and lactate release was quantified after 30 min (E). Data are mean ± *SEM (n=6).* Cytosol and mitochondria were prepared from C57Bl6 mice, and the fractions were immunoblotted with antibodies against VDAC, LDHA, and LDHB (F). The levels of LDHA and LDHB in the mitochondria are normalized to cytosolic LDHA and LDHB (G). The lactate efflux assay was carried out using mitochondrial preparation in the presence of glucose, phosphoenolpyruvate (PEP), and pyruvate (PEP), and control experiments were conducted with buffer instead of mitochondria (H). Data are mean ± *SEM (n=3).*

**Table S1: Real-time PCR primers to quantify the expression of *Ldha* and *Ldhb* in retina, rod photoreceptor, Cone, Müller, RGC, and RPE cells**

| **Gene** | **Forward Primer** | **Reverse Primer** |
| --- | --- | --- |
| *Ldha* | ACTGCAGGCTTCGATTACCC | ATGGACGTACACACTGGAGC |
| *Ldhab* | GGATTCACCCCGTGTCTACC | GAGCGACCTCATCGTCCTTC |
| *Rpl38* | CGCCATGCCTCGGAAA | CCGCCGGGCTGTCAG |
|  |  |  |

**Table S2. Antibodies used for immunofluorescence (IF) and immunoblot Analysis**

| **Antibody raised against** | **Host species** | **Dilution** | **Manufacturer** | **Catalog number** |
| --- | --- | --- | --- | --- |
| LDHA | Rabbit | 1:1000  1:50 (IF) | Proteintech | 21799-1-AP |
| LDHB | Rabbit | 1:1000  1:50 (IF) | Proteintech | 14824-1-AP |
| Phospho LDH (Tyr10) | Rabbit | 1:1000 | Cell Signaling Technology | 8176 |
| Pde6β | Mouse | 1:1000  1:25 (IF) | Santa Cruz | SC-377486 |
| Rhodopsin | Mouse | 1:1000  1:50 (IF) | In-house | Gift from Dr. Jim McGinnis (OUHSC) |
| Rod-Arrestin | Mouse | 1:1000  1:500 (IF) | In-house | Gift from Dr. Paul Hargrave (University of Florida) |
| Rod Transducin alpha (Trα) | Rabbit | 1:1000 | Santa Cruz Biotechnology | SC-389 |
| M-opsin | Rabbit | 1:1000  1:100 (IF) | Millipore Sigma | AB5405 |
| S-opsin | Rabbit | 1: 100 (IF) | Millipore Sigma | ABN1660-1 |
| Cone-Arrestin | Rabbit | 1:1000 | Millipore Sigma | AB15282 |
| Actin | Mouse | 1:1000 (IB) | Thermo Fisher Scientific | MA1-744 |
| Glutamine synthetase (GS) | Mouse | 1:1000  1:50 (IF) | Abcam | Ab64613 |
| Glial fibrillary acid protein (GFAP) | Rabbit | 1:100 (IF) | Dako | 20334 |
| PKM1 | Rabbit mAb | 1:1000  1:50 (IF) | Cell Signaling Technology | 7067 |
| PKM2 | Rabbit mAb | 1:1000  1:100 (IF) | Cell Signaling Technology | 4053 |
| Phospho-PKM2 (Tyr105) | Rabbit | 1:1000  1:50 (IF) | Cell Signaling Technology | 3827 |
| PDH | Rabbit | 1:1000 | Cell Signaling Technology | 3205 |
| Phospho-PDH (S293) | Rabbit | 1:1000 | Cell Signaling Technology | 37115 |
| Aldolase C | Mouse | 1:1000  1:100 (IF) | EnCor Biotechnology, Inc. | MCA-4A9 |
| GLUT1 | Rabbit | 1:1000  1:25 (IF) | Novus Biologicals | NB110-39113 |
